# The hidden burden of college life: Stress, sleep, and salivary biomarkers in students—A cross-sectional study

**DOI:** 10.1371/journal.pmen.0000717

**Published:** 2026-09-15

**Authors:** Hongyu Xu, Zhiqiang Yan, Xinyi Kong, Shuang Wu, Fei Shi, Qiong Ye, Zijin Li, Jia Lu, Juan Fang, Kexin Chen, Jiajun Bao, Yiyao Huang

**Affiliations:** 1 College of Science and Technology, Wenzhou-Kean University, Wenzhou, Zhejiang, China; 2 Dorothy and George Hennings College of Science, Mathematics and Technology, Kean University, Union, New Jersey, United States of America; 3 Department of Neurosurgery, Xijing Hospital, Fourth Military Medical University, Xi'an, Shaanxi, China; 4 The Key Laboratory of Aerospace Medicine, Ministry of Education, Fourth Military Medical University, Xi'an, Shaanxi, China; 5 Department of Neurosurgery, Second Affiliated Hospital of Anhui Medical University, Hefei, Anhui, China; PLOS: Public Library of Science, UNITED KINGDOM OF GREAT BRITAIN AND NORTHERN IRELAND

## Abstract

Globally, university students face substantial psychological pressures, and stress-related mental health disorders are recognized as major public health concerns. While biomarkers such as cortisol have been extensively studied, integrated analyses of neuroendocrine-immune-circadian interactions remain limited. In this cross-sectional study with two complementary components, psychometric assessments of 637 undergraduates revealed that 90.4% experienced moderate-to-high perceived stress. These stress levels correlated with fatigue (r = 0.41, *p* < 0.01) and poor sleep quality (r = 0.35, *p* < 0.01). A second component involved laboratory-based diurnal biomarker profiling of 33 high-stress and low-stress participants, revealing stress-dependent physiological patterns. Specifically, high stress was associated with sympathetic activation (α-amylase↑), HPA axis dysregulation (altered diurnal cortisol patterns), circadian disruption (melatonin↑), and mucosal immune activation (IgA ↑ , lysozyme↑). The coupling of α-amylase and lysozyme suggested neuro-immune coordination (r = 0.52, *p* < 0.001). These findings indicate that salivary biomarker profiles are associated with perceived stress. Sleep quality and fatigue warrant further investigation as potential intervention targets in future longitudinal studies.

## Introduction

Stress is a psychobiological response to perceived environmental threat [1]. Among undergraduates, stress arises from academic and social pressures that can impair cognitive resilience, academic performance, and increase dropout risk [2–4]. Dropout rates have risen, with a substantial proportion attributed to psychological concerns [5]. The World Health Organization estimated annual global productivity losses due to anxiety and depression at $1 trillion USD, a figure expected to rise [6].] Unmanaged stress can affect emotional and physical health, relationships, and quality of life, potentially creating a cycle of mental and academic decline [7,8]. The economic impact extends beyond individual well-being to institutional productivity [5]. Understanding the mechanisms linking stress to health outcomes in students is therefore a research priority.

Biomarkers are measurable characteristics that can be assessed objectively and reproducibly [9]. Perceived stress has been studied in relation to various physiological biomarkers. Cortisol, released by the adrenal glands in response to stress, is one of the most commonly examined [6–8]. As a primary mediator of mammalian stress adaptation, cortisol can be measured in blood, saliva, and hair, and shows altered patterns in chronic stress [10,11]. Chronic stress is associated with characteristic dysregulation patterns: (1) blunted diurnal rhythm, with reduced morning cortisol awakening response (CAR) and elevated nocturnal cortisol [12]; (2) hypocortisolism paradox, where initial hypercortisolemia progresses to adrenal exhaustion, flattening the cortisol slope [13]; and (3) decoupled reactivity, with exaggerated acute spikes despite basal hypocortisolism [14]. These alterations reflect HPA axis maladaptation, with clinical implications for immune and metabolic health [15].

Recent research has advanced understanding of stress physiology through distinct biomarker signatures across interconnected systems. Salivary α-amylase is a marker of sympathetic-adrenal-medullary (SAM) axis activation, rising rapidly via β-adrenergic signaling during acute stress to promote glycogen breakdown for energy mobilization [16,17]. Chromogranin A (CHGA), co-released with catecholamines from adrenal chromaffin cells, is involved in hormone storage and release; its proteolytic fragments influence autonomic function, complementing cortisol measurements [18,19].

Secretory immunoglobulin A (sIgA), produced by submucosal plasma cells, is suppressed by glucocorticoids during chronic stress, reducing epithelial translocation and compromising mucosal defense [19,20]. Lysozyme (LZM), an antimicrobial enzyme, shows dynamic stress-related changes and serves as an indicator of innate immune capacity [21]. Melatonin, synthesized by pinealocytes under suprachiasmatic nucleus (SCN) regulation, undergoes secretion phase shifts due to stress-induced corticotropin-releasing hormone (CRH), disrupting circadian entrainment of sleep-wake cycles via MT1/MT2 receptor downregulation [22]. This integrated biomarker panel—spanning neuroendocrine (α-amylase, CHGA), immune (sIgA, LZM), and circadian (melatonin) domains—allows multidimensional stress assessment. Stress physiology involves crosstalk among neuroendocrine, immune, and circadian systems [23,24]. Acute stress triggers synchronous SAM-HPA activation: norepinephrine stimulates α-amylase within minutes, while CRH initiates cortisol secretion [25]. However, sustained stress disrupts this coordination: chronic SAM activation can deplete HPA responsiveness, flattening cortisol rhythms despite persistent α-amylase elevation [26]. Melatonin phase shifts may desynchronize glucocorticoid oscillations and amplify nocturnal immunosuppression, potentially creating a cycle where disrupted sleep impairs stress recovery [27]. Thus, these biomarkers may reflect key points of dysregulation in stress pathophysiology.

The cyclical interaction between psychological stress, fatigue, and sleep disturbances is recognized as a pathway to mental health decline, though its mechanisms remain incompletely understood [28]. Evidence indicates that elevated stress impairs sleep and amplifies fatigue, creating a self-perpetuating cycle. While interventions targeting these factors show promise, progress has been limited by methodological constraints in capturing real-time psychobiological dynamics [29–31]. The stress-sleep relationship is bidirectional and complex. As highlighted by Gardani et al. (2022), stress can precipitate sleep difficulties through multiple mechanisms [12]. To address this gap, the present study employed a non-invasive psychobiological profiling approach using salivary biomarkers. This approach may help identify preclinical risk signatures and support early intervention [32].

Student stress is multifaceted, involving psychological, social, and environmental factors—including academic overload, developmental transitions, and social pressures [33,34]. Previous studies on undergraduate stress have been limited by reliance on either subjective self-reports or isolated biomarkers (e.g., cortisol alone), hindering a comprehensive understanding of how stress affects neuroendocrine, immune, and circadian systems simultaneously. The current study addressed this gap through an integrative cross-sectional design, combining validated questionnaires (PSS, PSQI, FSS) with parallel analyses of six salivary biomarkers: α-amylase (SAM axis), cortisol (HPA axis), melatonin (circadian regulation), IgA, lysozyme, and chromogranin A [13].

This study aimed to determine the prevalence of perceived stress, fatigue, and poor sleep quality among undergraduates, and to identify biomarkers associated with stress levels. Guided by psychophysiological models, we hypothesized that high perceived stress would be associated with: (a) elevated salivary α-amylase (SAM-axis hyperactivity) and cortisol (HPA-axis activation); and (b) disruption of circadian-immune biomarkers (melatonin phase shift, alterations in IgA and lysozyme). Specifically, we aimed to: (1) quantify the prevalence and interrelationships of perceived stress, fatigue, and poor sleep quality; (2) identify salivary biomarkers associated with heightened stress; and (3) characterize the physiological signature of stress in this population. This characterization may inform the selection of targets for future hypothesis-driven longitudinal studies and evidence-based interventions [35].

## Materials and methods

### Ethics Statement

The study protocol was approved by the Ethics Committee of Wenzhou-Kean University (Approval No. WKUIRB2023–008) and conducted in accordance with the WMA Declaration of Helsinki. All participants provided written informed consent after being informed of potential risks and benefits.

### Participants

Participants maintained their regular eating, studying, and exercising routines. Except for coffee, carbonated beverages, and similar items, dietary intake was unrestricted. A total of 675 undergraduates were initially enrolled between March 3 and May 5, 2023. After excluding 38 incomplete responses, 637 participants (mean age 19.85 years, SD = 1.45) were included. Recruitment and questionnaire data collection occurred over three weeks within this period. Participants were recruited via email advertisements; participation was voluntary and confidential. Sessions included approximately 60 participants who completed questionnaires within 30 minutes starting at 3:00 PM in a classroom under the supervision of three certified evaluators [36]. The 3:00 PM time was chosen to minimize diurnal mood variations that could bias responses [16].

**Fig 1:**
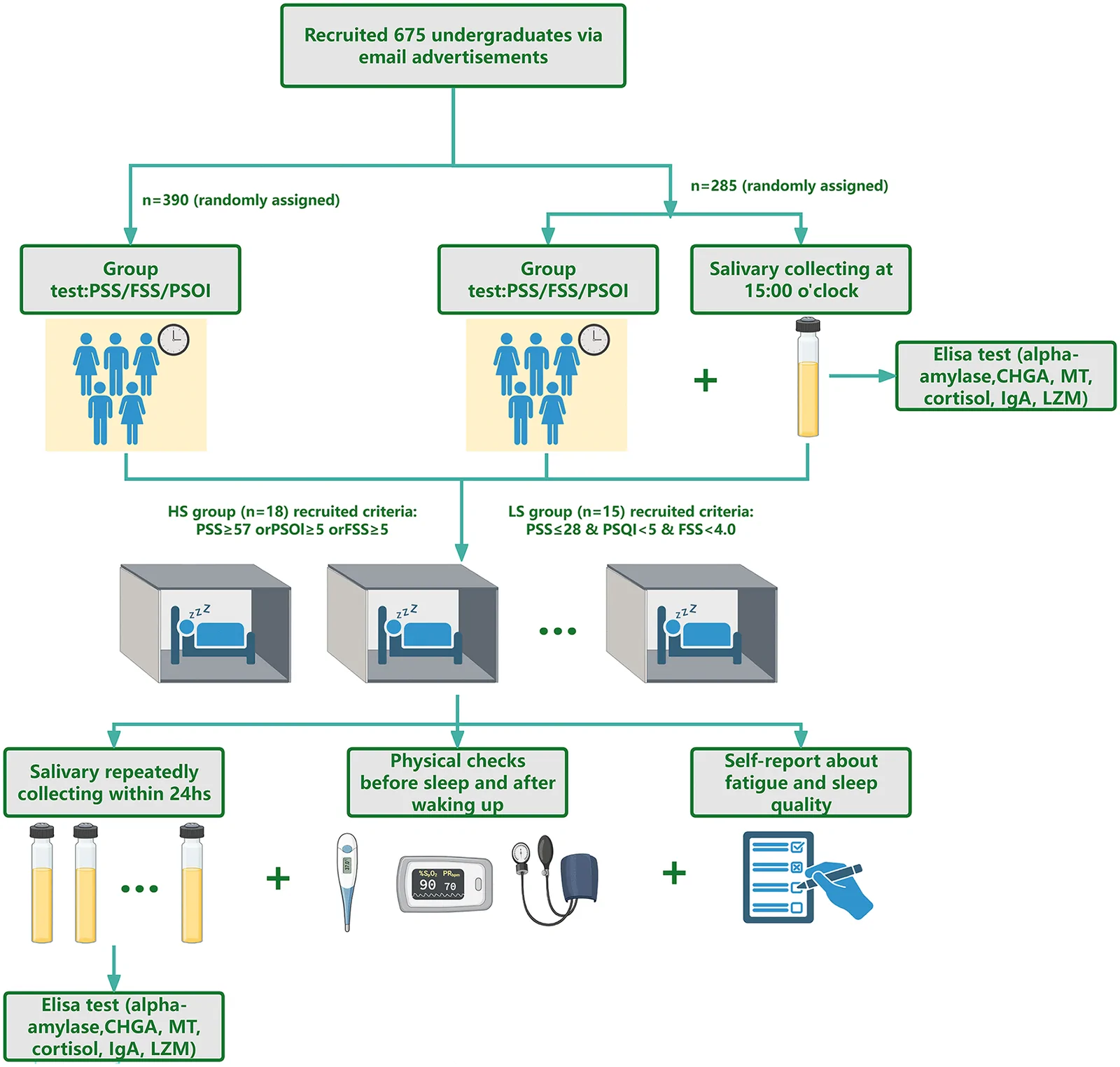
Overview of the study design and methodology. Created with BioRender. Wong, **A.** (2026). https://BioRender.com/i61e911.

A random subset of 285 students was selected for biomarker analysis, with saliva collected at 3:00 PM. Of these, 23 samples were excluded due to insufficient volume or poor quality, leaving 262 samples for ELISA. Post-hoc power analysis indicated that with α = 0.05, the sample size of 637 for questionnaire analyses and 33 for diurnal profiling provided > 80% power to detect medium-to-large effect sizes (Cohen’s d ≥ 0.5) (Fig 1).

### Instruments and data collection

The questionnaire packet included the PSS, FSS, PSQI, and a demographic survey covering age, gender, academic background, medication use, stress exposure, alcohol use, and gaming behavior. All assessments were administered in Chinese.

The PSS is a 14-item scale measuring perceived stress on a 5-point Likert scale (0 = never, 4 = very often). Total scores range from 14 to 70, with higher scores indicating greater perceived stress: low (14–28), moderate (29–42), high (43–56), and very high (57–70). The internal consistency of the PSS-14 was evaluated Cronbach’s alpha coefficient, yielding a reliability score of 0.78 for the overall scale [37,38].

The FSS is a 9-item scale assessing fatigue severity on a 7-point Likert scale (1 = strongly disagree, 7 = strongly agree). The total score is the mean of the nine items, with higher values reflecting greater fatigue [19]. Internal consistency was α = 0.88, and test-retest reliability was r = 0.84 [17].

The PSQI assessed subjective sleep quality over the preceding month [20]. This 19-item instrument yields seven component scores (subjective sleep quality, sleep latency, sleep duration, habitual sleep efficiency, sleep disturbances, use of sleep medication, and daytime dysfunction), each scored 0–3, with a global score ranging from 0 to 21 [21,39]. Following established criteria, participants with PSQI > 5 were classified as having poor sleep quality [23]. Internal consistency, as measured by Cronbach's α, was 0.83 in this sample.

### Participants for diurnal sample collection

Following questionnaire screening, 15 participants met the low-stress (LS) criteria (PSS ≤ 28, PSQI <5, and FSS < 4.0; all three required), and 18 met the high-stress (HS) criteria (at least one of: PSS ≥ 57, PSQI ≥ 5, or FSS ≥ 5). Inclusion criteria were: (1) no history of psychiatric or other diseases; (2) no long-term medication use. Initially, 40 participants met at least one HS criterion; of these, 22 were excluded due to ongoing psychotropic medication use (e.g., sertraline, trazodone), leaving 18 participants in the final HS group. All participants provided written informed consent and participation was voluntary and confidential.

### Diurnal sample collection protocol

A total of 33 participants were accommodated in dedicated rooms at Wenzhou-Kean University (Wenzhou, China) for a 24‑h study period. Participants checked in on the evening prior to the experiment and were permitted to leave after the final sample collection. Outside the designated sampling times, they were allowed to engage in their usual activities.

EP tubes were stored in foil to protect samples from light. A designated experimenter collected samples at each time point, ensuring that melatonin and cortisol samples were obtained immediately upon waking.

Participants followed these pre-sampling guidelines: (1) no alcohol or caffeine 24 hours before; (2) no food or drink 1 hour before each session; (3) no strenuous exercise 3 hours before. All participants woke at 8:30 AM and rinsed their mouths with water. Saliva samples were collected at 9:00, 9:30, 10:00, 11:00, 12:00, 14:00, 16:00, 17:00, 18:00, 20:00, and 23:00 under technician supervision to minimize contamination. Participants provided 2 ml of saliva by passive drool into sterile tubes; samples were immediately placed on ice and stored at −80°C. Compliance was monitored through direct observation.

To minimize confounders, participants’ physical condition, stress, and fatigue were monitored. Demographic information (gender, height, weight) was recorded. Body temperature, blood pressure, and oxygen saturation were measured each morning and evening. Upon waking, participants completed a self-assessment form covering bedtime, sleep onset, wake time, sleep quality, and fatigue severity.

### ELISA

Saliva samples were thawed on ice, centrifuged at 1,000 × g for 20 min at 4 ℃, and the supernatant was stored at -80 ℃. α-amylase, CHGA, IgA, and lysozyme were measured using sandwich ELISA, and cortisol and melatonin using competitive ELISA, following the manufacturer's protocols (Elabscience, Houston, TX, USA). Intra-assay precision was assessed by testing three samples (low, mid, and high concentrations) 20 times on a single plate; inter-assay precision was assessed by analyzing the same samples on three separate plates, with 20 replicates per plate.

### Statistical analysis

Data analysis included: (1) descriptive statistics; (2) Pearson correlations among PSS, FSS, PSQI, and salivary biomarkers (Spearman for non-normal variables); (3) one-way ANOVA for group differences (gender, grade, major) with LSD post-hoc; (4) two-way ANOVA for gender × stress effects on PSQI and FSS; (5) repeated-measures ANOVA and generalized estimating equations (GEE) for time × group effects on biomarkers (log transformation for non-normal data); and (6) Random Forest machine learning (70% training/30% test) with GridSearchCV and 5-fold cross-validation. Prevalence rates used Wilson score 95% CIs; group differences used χ² tests.

Significance thresholds were *p* < 0.05, *p* < 0.01, and *p* < 0.001. Analyses used SPSS 26.0 and Python. Mauchly's test and Shapiro-Wilk test were applied; Greenhouse-Geisser correction was used when sphericity was violated.

## Results

### General perceived stress and fatigue state of the population

Of 675 invited participants, 637 (mean age 19.85 years, SD = 1.45) completed the questionnaires (response rate 94.1%); 38 were excluded due to incompleteness. The final sample included 217 men and 420 women: 38.1% freshmen, 24.2% sophomores, 21.0% juniors, and 16.6% seniors. Psychotropic drug use was reported by 23 (3.6%), 52 (8.2%) came from single‑parent families, and 174 (27.3%) had experienced traumatic events in the past six months (S1 Table).

**Fig 2:**
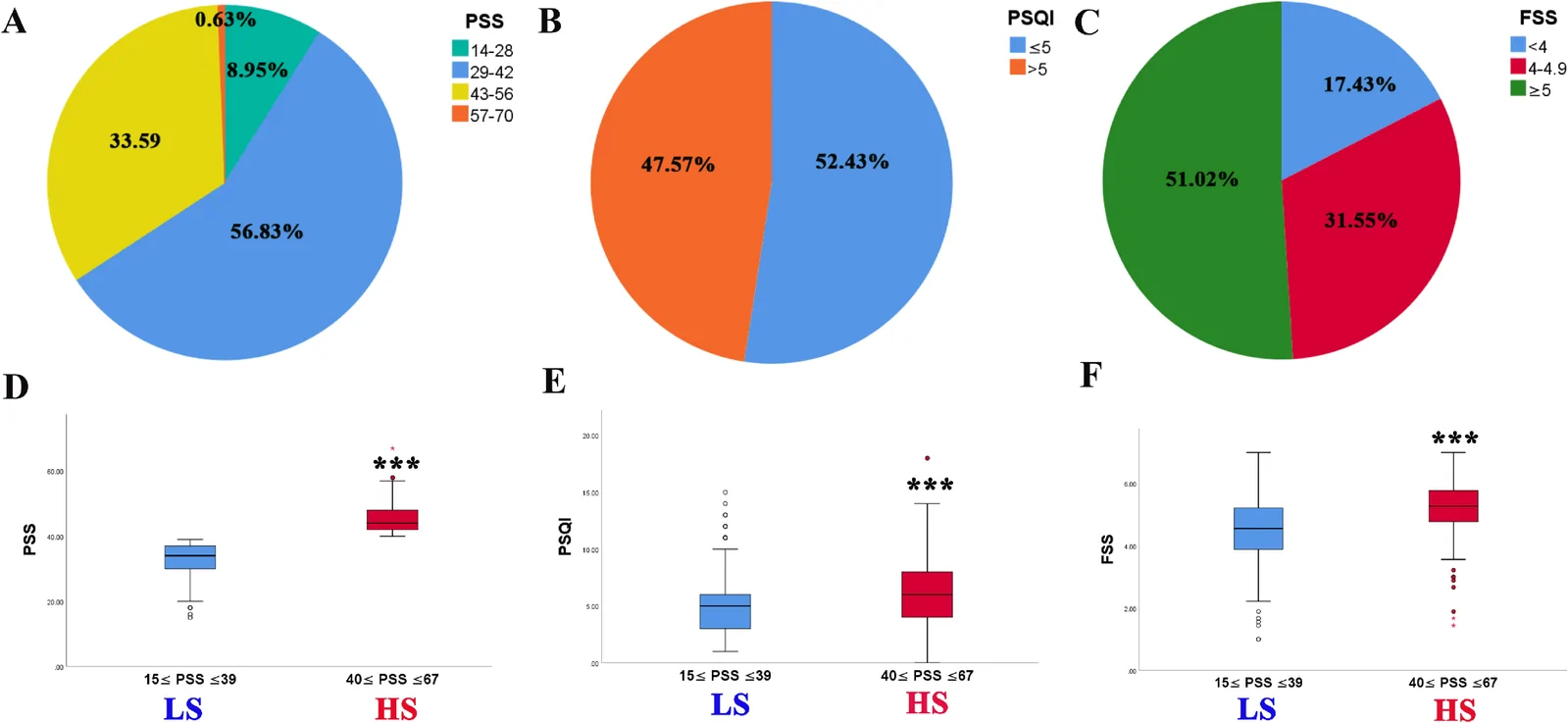
Distribution of scores for the Perceived Stress Scale (PSS) (A), Pittsburgh Sleep Quality Index (PSQI) (B), and Fatigue Severity Scale (FSS) (C) among 637 students. PSS categories were defined as low stress (14–28), moderate stress (29–42), high stress (43–56), and very high stress (57–70). Participants with PSQI > 5 were classified as having poor sleep quality. For the FSS, moderate fatigue was defined as 4–4.9 and severe fatigue as ≥ 5. Participants were divided into two groups based on PSS scores using K‑means clustering. A highly significant difference between the two groups was found (*p* < 0.001, **D)**. Students with higher PSS scores also showed significantly higher PSQI (*p* < 0.001, E) and FSS (*p* < 0.001, F) scores. Although the box plots in panels E and F show some overlap in score distributions, differences in group means were statistically significant, as confirmed by two-way ANOVA. No significant gender differences were observed between the two groups.

Mean scores were: PSS = 39.18 (SD = 7.51), FSS = 4.87 (SD = 1.05), and PSQI = 5.61 (SD = 2.71), with no significant gender differences (PSS: F = 0.87, *p* = 0.35; FSS: F = 0.04, *p* = 0.83; PSQI: F = 0.06, *p* = 0.807). Moderate perceived stress was reported by 56.83% and high stress by 33.59% (Fig 2A). Poor sleep quality (PSQI > 5) was found in 47.57% (Fig 2B); PSQI scores ranged from 2 to 18. Fatigue was moderate (FSS 4-4.9) in 31.55% and severe (FSS ≥ 5) in 51.02% (Fig 2C).

As shown in S2 Table, MANOVA with LSD post-hoc tests revealed that freshmen had significantly lower FSS scores than other grades, indicating less fatigue. Sophomores had higher PSS scores than freshmen (*p* = 0.03), but not significantly different from juniors (*p* = 0.15) or seniors (*p* = 0.13). For PSQI, sophomores showed higher total scores and greater subjective sleep quality impairment than juniors (*p* < 0.05) and freshmen (*p* < 0.01), as well as greater sleep latency than freshmen and seniors, and the highest daytime dysfunction among all grades (*p* < 0.01). Both sophomores (*p* < 0.01) and seniors (*p* < 0.01) had greater sleep latency than freshmen.

Stress prevalence across academic years was: low 8.95%, moderate 56.83%, high 33.59%, and very high 0.63%, with no significant grade differences (χ² = 6.87, *p* = 0.65). Although sophomores had higher mean PSS scores than freshmen, the distribution across stress categories did not differ significantly by grade.

### Perceived stress-related factors

PSS correlated positively with PSQI (r = 0.35, *p* < 0.01) and FSS (r = 0.41, *p* < 0.01); FSS also correlated with PSQI (r = 0.37, *p* < 0.01) (S3 Table). K-means clustering divided participants into LS (PSS 15–39) and HS (PSS 40–67) groups, with a significant group difference (Fig 2D, F = 1020.91, *p* < 0.001). Two-way ANOVA showed HS had higher FSS (mean 5.21 vs. 4.53, F = 68.74, *p* < 0.001; Fig 2E) and PSQI (mean 6.36 vs. 5.09, F = 28.22, *p* < 0.001; Fig 2F) than LS. No significant gender differences were found.

**Fig 3:**
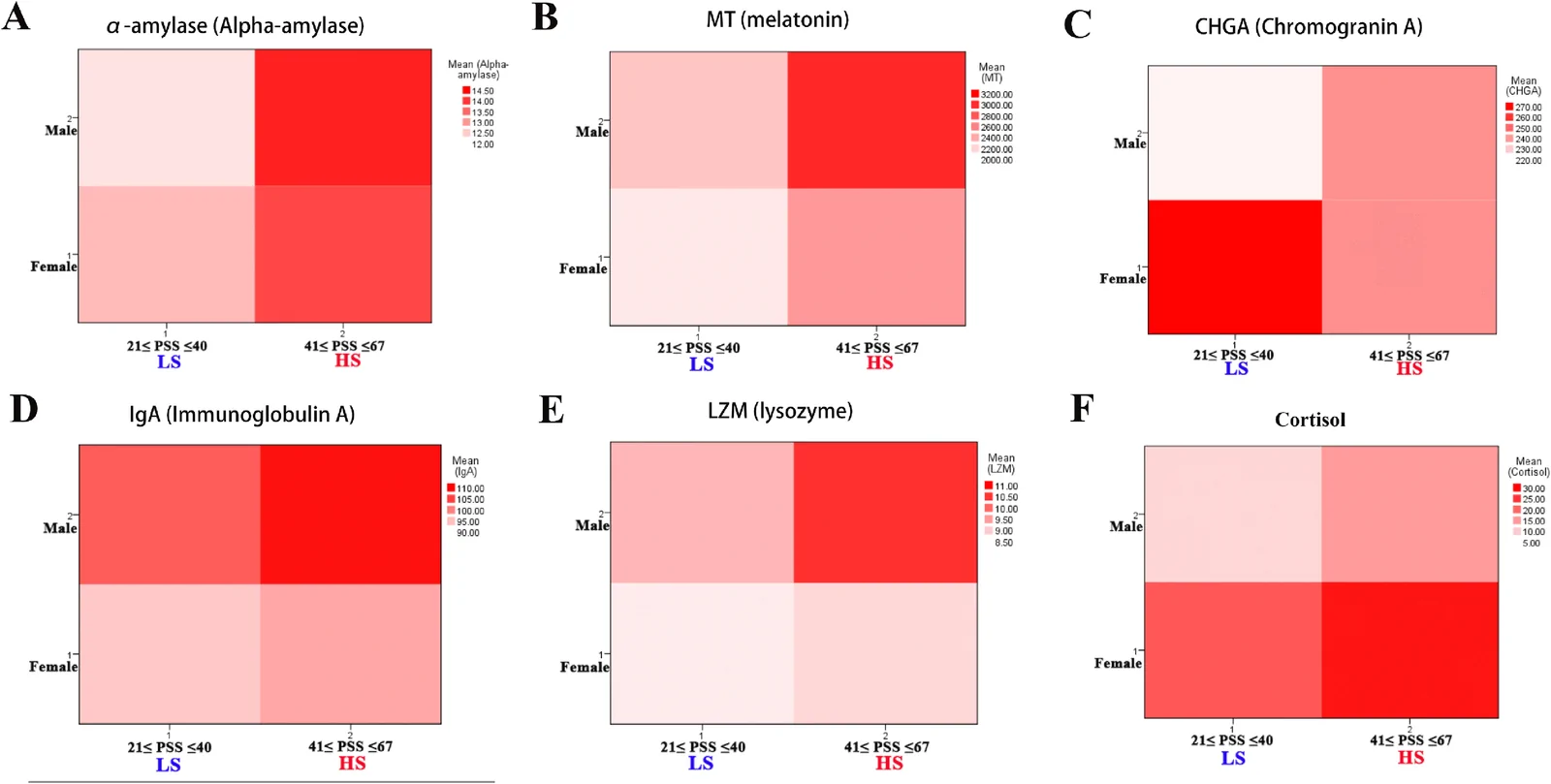
Heat map illustrating the mean concentrations of salivary biomarkers: alpha-amylase (A, μg/ml), melatonin (MT; B, pg/ml), chromogranin A (CHGA; C, ng/ml), immunoglobulin A (IgA; D, μg/ml), lysozyme (LZM; E, μg/ml), and cortisol (F, μg/dl). Groups were stratified by PSS score and gender. N = 262.

**Fig 4:**
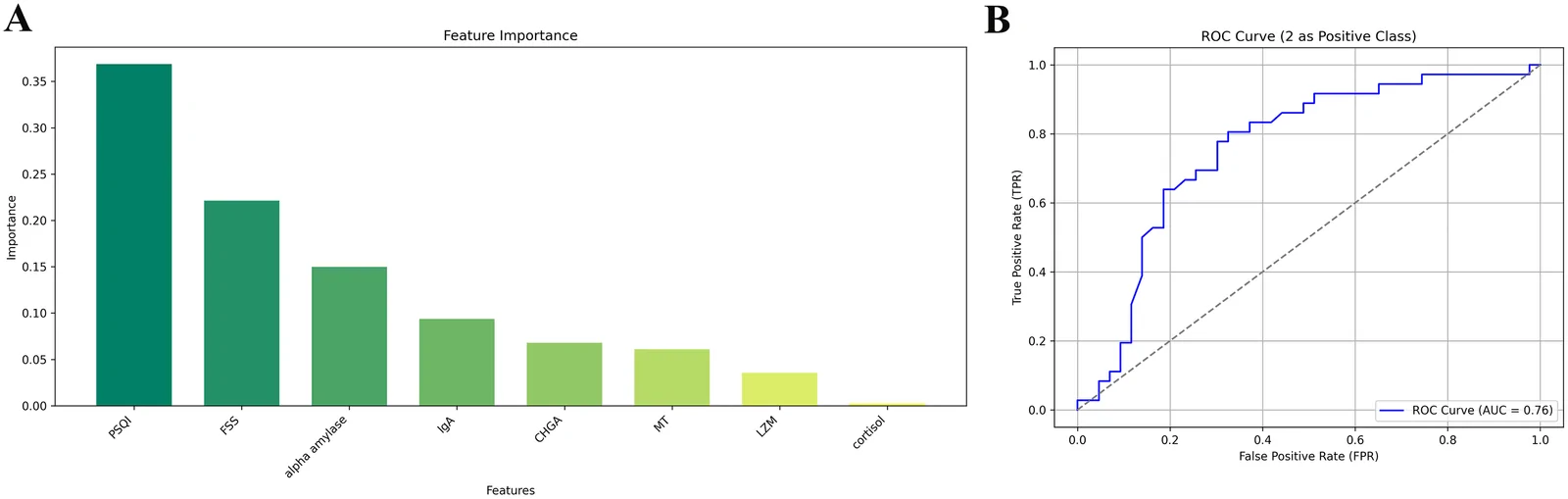
Machine learning analysis was conducted to assess the severity of perceived stress. Panel (A) shows the feature importance of various factors, while the receiver operating characteristic (ROC) curve (B) indicates an area under the curve (AUC) of 0.76. N = 262.

Saliva samples from 262 participants (3 PM) were analyzed for α-amylase, MT, CHGA, IgA, lysozyme, and cortisol; 23 samples were excluded due to poor quality. K-means clustering re-divided participants into LS (PSS 21–40) and HS (PSS 41–67). As shown in Fig 3, HS had higher levels of α-amylase (A), MT (B), IgA (D), lysozyme (E), and cortisol (F) than LS, but not CHGA (C). Females generally had lower α-amylase, MT, IgA, and lysozyme but higher cortisol; no consistent gender pattern was observed for CHGA. In an exploratory random-forest analysis (AUC = 0.76, 95% CI: 0.71-0.81), permutation importance (bootstrap, n = 1000) identified FSS (0.35, 95% CI: 0.30-0.40) and PSQI (0.21, 95% CI: 0.17-0.25) as the top predictors, followed by α-amylase (0.14, 95% CI: 0.10-0.18); model accuracy was 76% (Fig 4). Feature importance values were consistent: FSS (0.37), PSQI (0.22), and α-amylase (0.15) were most strongly associated with PSS, suggesting a potential role for these biomarkers in stress prediction that warrants further investigation.

### The relationships among concentrations of salivary α-amylase, MT, CHGA, IgA, LZM, and cortisol

Pearson correlations among α-amylase, MT, CHGA, IgA, lysozyme, and cortisol are shown in S4 Table (N = 262). α-amylase showed statistically significant but very weak positive correlations with IgA (r = 0.14, *p* < 0.05), cortisol (r = 0.14, *p* < 0.05), and MT (r = 0.14, *p* < 0.05), likely attributable to the large sample size. In contrast, α-amylase was strongly correlated with lysozyme (r = 0.52, *p* < 0.001). MT concentrations showed positive correlations with IgA (r = 0.29, *p* < 0.001) and cortisol (r = 0.20, *p* < 0.01), and negatively with lysozyme (r = -0.18, *p* < 0.01).

**Fig 5:**
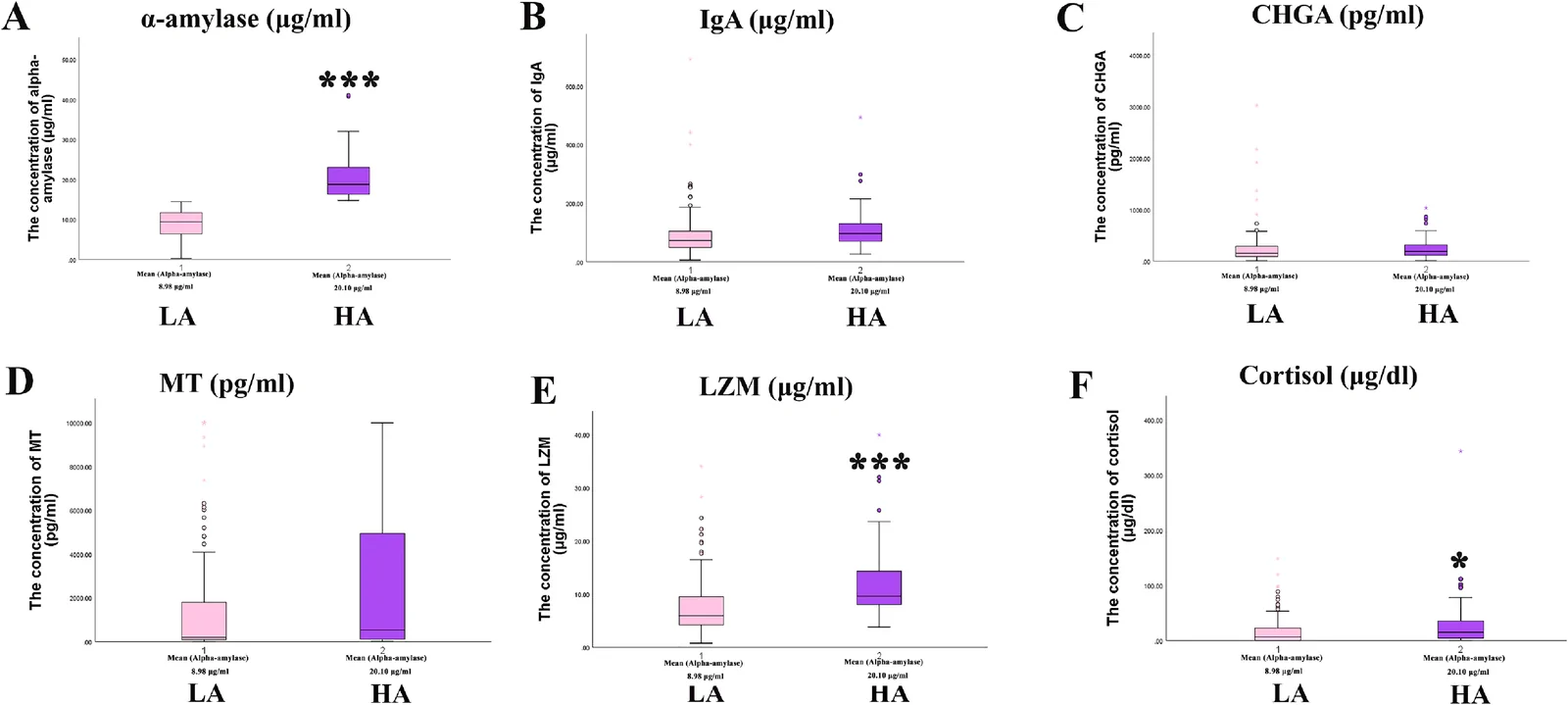
Box plots showing the distribution of salivary biomarker concentrations stratified by alpha-amylase level (LA vs. HA). Because no significant group-by-gender interaction was detected, data from male and female genders were combined. (A) alpha-amylase (μg/ml), **(B)** IgA (μg/ml), **(C)** CHGA (pg/ml), **(D)** MT (pg/ml), (E) lysozyme (μg/ml), and (F) cortisol (μg/dl). N = 262. Significance markers indicate comparisons between LA and HA groups (*p < 0.05, ***p < 0.001).

K-means clustering classified participants into low (LA; mean ± SD = 8.98 ± 3.32 μg/ml) and high (HA; 20.10 ± 5.06 μg/ml) α-amylase groups. Two-way ANOVA showed HA had higher lysozyme (11.72 ± 5.91 vs. 7.50 ± 5.09 μg/ml; F = 30.01, *p* < 0.001; Fig 5E) and cortisol (27.49 ± 41.68 vs. 16.28 ± 24.57 μg/dl; F = 6.78, *p* < 0.05; Fig 5F) than LA. No significant differences were observed for IgA (F = 2.45, *p* = 0.12; Fig 5B), MT (F = 1.89, *p* = 0.17; Fig 5D), or CHGA (F = 0.15, *p* = 0.70; Fig 5C). No group-by-sex interactions were detected (all *p* > 0.05). Cortisol was higher in women than men (F = 8.71, *p* < 0.01).

### The diurnal course differences of salivary α-amylase, LZM, cortisol, MT, IgA, and CHGA

Diurnal profiles of α-amylase, lysozyme, cortisol, MT, IgA, and CHGA were assessed at 11 time points (9:00, 9:30, 10:00, 11:00, 12:00, 14:00, 16:00, 17:00, 18:00, 20:00, 23:00) in 33 participants: HS (n = 18, 8 men/10 women; PSS ≥ 57, PSQI ≥ 5, or FSS ≥ 5) and LS (n = 15, 6 men/9 women; PSS ≤ 28, PSQI < 5, and FSS < 4). No within-group gender differences were detected (*p* > 0.05).

**Fig 6:**
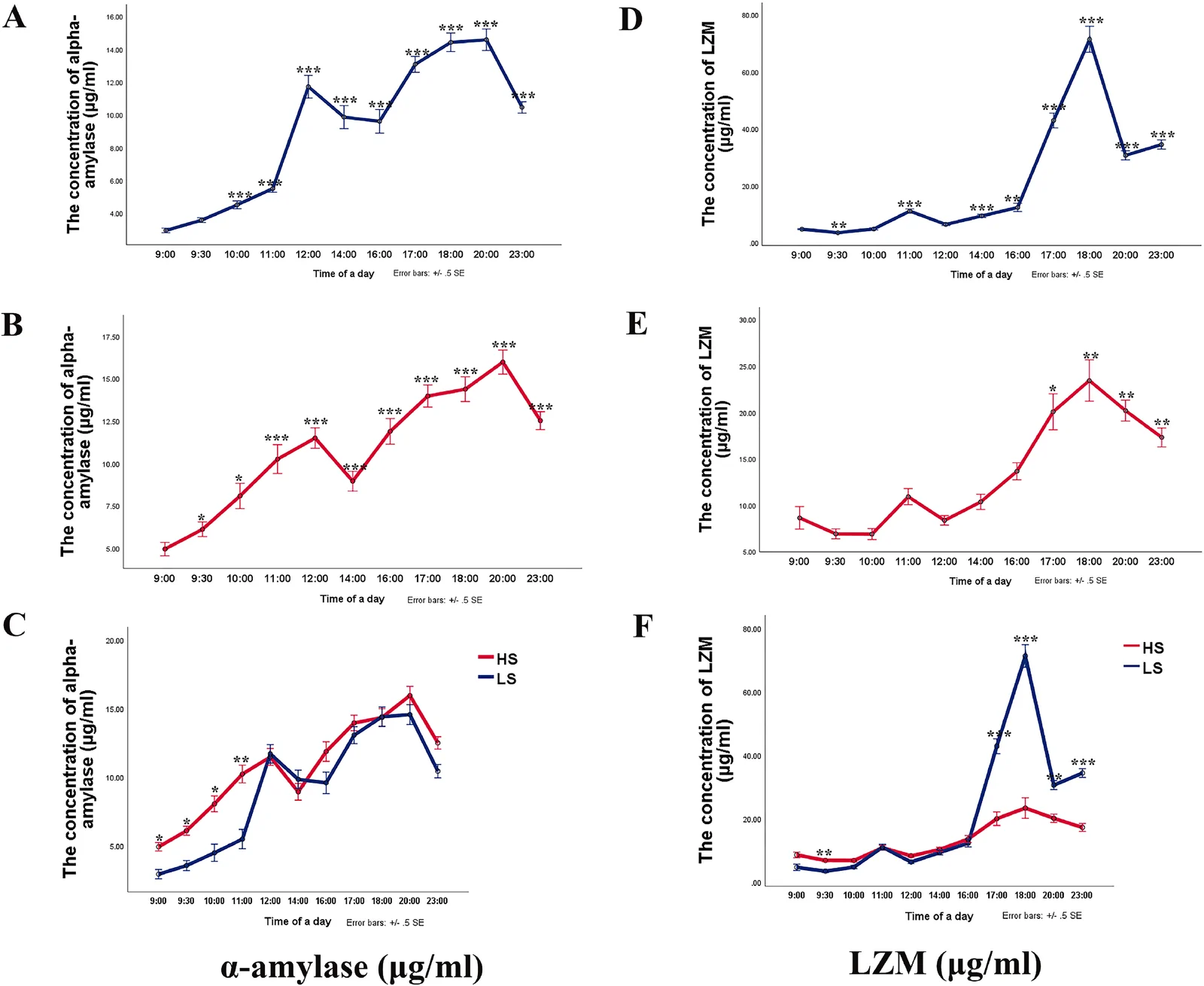
Diurnal profiles of salivary alpha-amylase (A–C) and lysozyme (LZM; D–F) measured at 11 time points: 9:00 (wake-up), 9:30, 10:00, 11:00, 12:00, 14:00, 16:00, 17:00, 18:00, 20:00, and 23:00 (bedtime). Participants were stratified into high-stress (HS, n = 18) and low-stress (LS, n = 15) groups based on questionnaire scores. The HS group met at least one of the following criteria: PSS ≥ 57, PSQI ≥ 5, or FSS ≥ 5. The LS group met all three criteria: PSS ≤ 28, PSQI < 5, and FSS < 4. Significance levels: *p < 0.05, **p < 0.01, ***p < 0.001.

α-amylase (Fig 6A–C): HS showed a significant rise within 30 min of waking; LS showed a similar rise after ~1 h. Both groups exhibited a similar pattern of alpha-amylase fluctuation over time: alpha-amylase level steadily increased: increasing until noon, dipping at 14:00, peaking at 20:00, then declining by 23:00. GEE confirmed significant time and group effects (Fig 6C). HS had higher levels than LS at 9:00, 9:30, 10:00, and 11:00, but levels converged by noon, with no differences at other time points.

Lysozyme (Fig 6D–F): In LS, levels gradually rose from 9:30–16:00, sharply increased at 17:00, peaked at 18:00, then declined at 20:00. In HS, a significant rise occurred only after 17:00, also peaking at 18:00. HS had higher lysozyme at 9:30, whereas LS had higher levels at 17:00, 18:00, 20:00, and 23:00 (Fig 6F).

**Fig 7:**
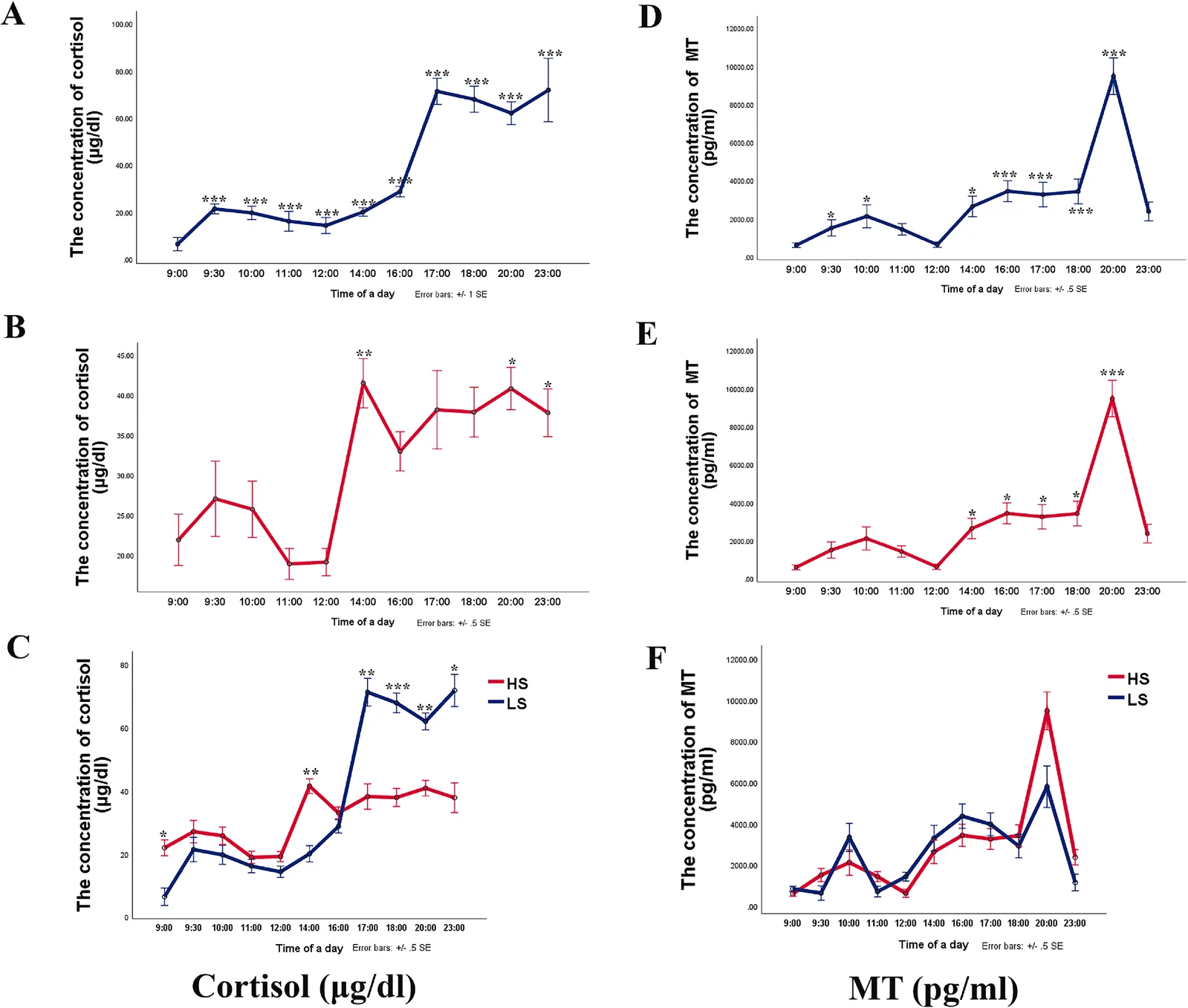
Diurnal profiles of salivary cortisol (G–I) and melatonin (MT; J–L) measured at the same 11 time points: 9:00 (wake-up), 9:30, 10:00, 11:00, 12:00, 14:00, 16:00, 17:00, 18:00, 20:00, and 23:00 (bedtime). Participants were stratified into high-stress (HS, n = 18) and low-stress (LS, n = 15) groups based on questionnaire scores. The HS group met at least one of the following criteria: PSS ≥ 57, PSQI ≥ 5, or FSS ≥ 5. The LS group met all three criteria: PSS ≤ 28, PSQI < 5, and FSS < 4. Significance levels: *p < 0.05, **p < 0.01, ***p < 0.001.

Cortisol (Fig 7A–C): LS showed a clear diurnal pattern: rising 30 min after waking, stable until 16:00, peaking at 17:00, and remaining elevated until bedtime. HS showed greater variability, with a significant rise only at 14:00, followed by fluctuations and elevated levels after 20:00. HS had higher cortisol at 9:30 and 14:00; LS had higher levels from 17:00–23:00 (Fig 7C).

MT (Fig 7D–F): Both groups showed similar patterns: higher levels after 14:00, peaking at 20:00, then declining by bedtime. LS also showed increases at 9:30 and 10:00. No significant group differences were observed at any time point (Fig 7F).

**Fig 8:**
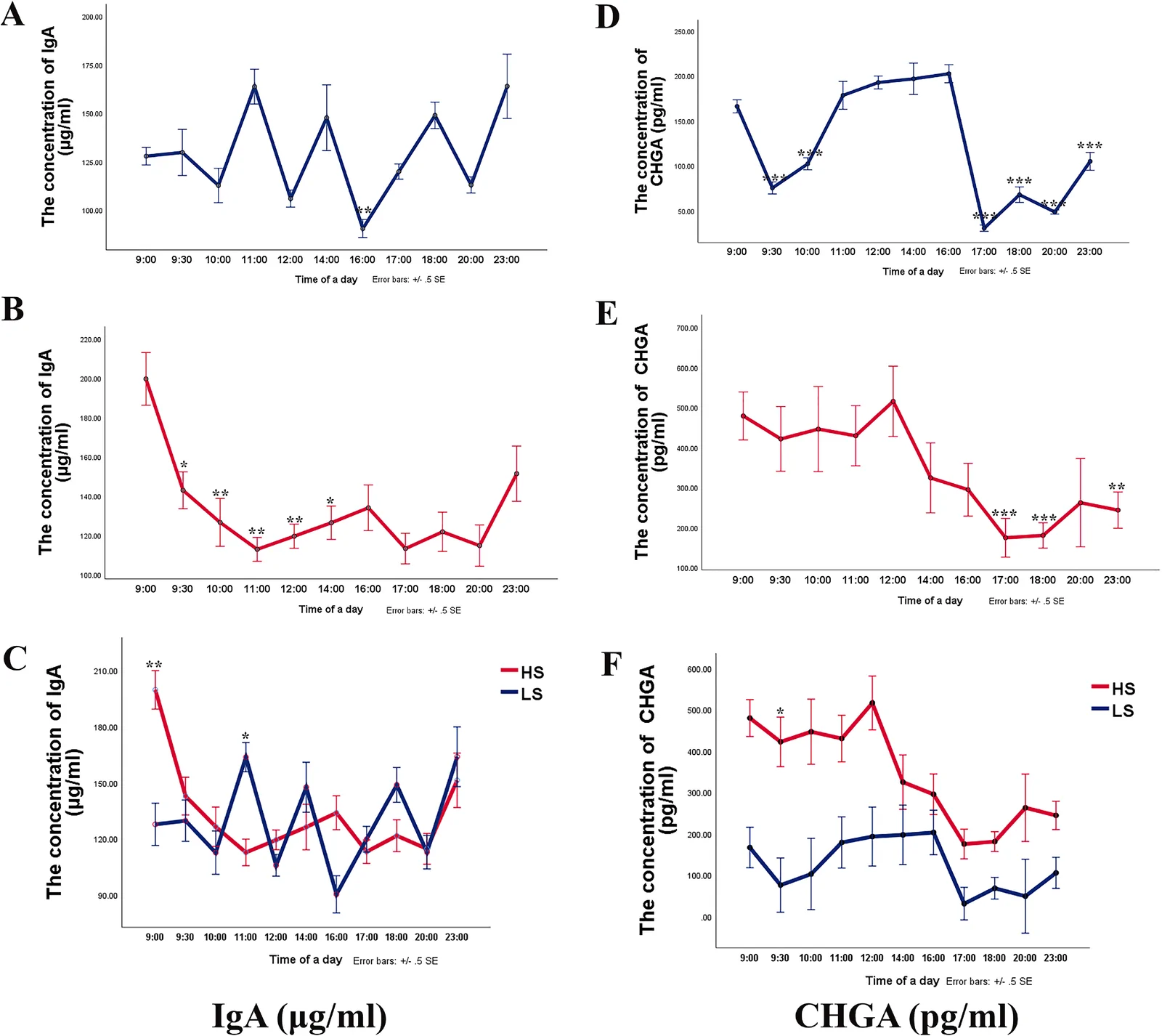
Diurnal profiles of salivary IgA (M–O) and chromogranin A (CHGA; P–R) measured at the same 11 time points: 9:00 (wake-up), 9:30, 10:00, 11:00, 12:00, 14:00, 16:00, 17:00, 18:00, 20:00, and 23:00 (bedtime). Participants were stratified into high-stress (HS, n = 18) and low-stress (LS, n = 15) groups based on questionnaire scores. The HS group met at least one of the following criteria: PSS ≥ 57, PSQI ≥ 5, or FSS ≥ 5. The LS group met all three criteria: PSS ≤ 28, PSQI < 5, and FSS < 4. Significance levels: *p < 0.05, **p < 0.01, ***p < 0.001.

IgA (Fig 8A–C): IgA showed more variability than other biomarkers. LS had no consistent pattern, with significantly lower levels at 16:00 than at 9:00 (Fig 8A). HS peaked at 9:00, sharply declined by 9:30, reached the lowest level at 11:00, and returned to morning levels after 16:00 (Fig 8B). GEE showed HS had higher IgA at 9:00, while LS had higher levels at 11:00 (Fig 8C).

CHGA (Fig 8D–F): In LS, CHGA decreased after waking, rose 1 h later, stabilized between 11:00 and 16:00, then sharply declined to the lowest level at 17:00. In HS, CHGA steadily decreased until 17:00. GEE showed HS had higher CHGA at 9:30, with no other group differences (Fig 8F).

## Discussion

Psychological stress is an important concern for undergraduates, as it can contribute to anxiety and depression, with consequences for health and societal productivity. Mental health disorders impose significant economic burdens through healthcare costs and lost productivity, yet stigma and limited access hinder help‑seeking [24,25]. This study examined the relationships among perceived stress, fatigue, and sleep quality in undergraduates using validated questionnaires and salivary biomarkers. The cross-sectional design allowed simultaneous assessment of subjective and objective stress indicators, capturing integrated psychobiological profiles that could inform targeted interventions. The observed correlations among perceived stress, fatigue, sleep quality, and salivary biomarkers suggest that such biomarkers may hold potential for guiding intervention strategies.

To our knowledge, this is among the first studies to systematically measure six salivary biomarkers—α-amylase, cortisol, melatonin, IgA, lysozyme, and chromogranin A—in parallel within a student population. Unlike earlier work that focused on single biomarkers (e.g., cortisol or α-amylase alone), this multi-metric approach provides a broader view of stress-related changes across neuroendocrine (SAM vs. HPA axes), immune (mucosal and antimicrobial), and circadian domains. Integrating data across these systems adds to the evidence supporting a network-based conceptualization of stress physiology. However, the cross-sectional nature of this study precludes causal interpretations, and longitudinal research is needed to clarify whether biomarker alterations precede or follow psychological distress.

The overall stress prevalence in this sample (moderate: 56.83%; high: 33.59%; total: 90.42%) was higher than rates reported in other student populations, such as 52.7% among Moroccan medical students [40] and 54–55% among medical students in Bangladesh [26]. Stress levels also varied by academic year: sophomores reported higher stress than freshmen, consistent with findings from Chinese medical students [41], though other studies have noted elevated stress in first-year students, attributed to academic, social, and financial transitions [42,43]. These grade-related differences likely reflect varying academic demands, social support, and adaptive capacity over the course of college, underscoring the importance of considering these factors when interpreting stress patterns across academic years.

While most cross-biomarker correlations were modest, α-amylase and lysozyme showed a robust association (r = 0.52), suggesting coordinated sympathetic-immune activation. α-amylase, a marker of SAM axis activity, is released via β-adrenergic signaling during acute stress; lysozyme, an innate immune enzyme, is similarly modulated by sympathetic activation through norepinephrine-stimulated neutrophil degranulation and NF-κB signaling. Their coupling may reflect enhanced oral mucosal antimicrobial defenses under stress. This α-amylase-lysozyme pair warrants further investigation as a candidate biomarker combination for non-invasive stress monitoring, though clinical applications would require additional validation. The cortisol profile in HS students—characterized by a blunted morning rise, a peak at 14:00, and elevated nocturnal levels—deviated from typical diurnal patterns. Prolonged stress may blunt HPA axis reactivity, flattening circadian cortisol rhythms through impaired negative feedback [27]. The 14:00 peak may represent a compensatory surge after morning HPA hypoactivation, consistent with reports linking chronic stress to altered cortisol pulsatility [44,45]. This pattern is consistent with a stress-induced HPA dysregulation phenotype. In addition, although melatonin secretion is normally minimal during daytime and further suppressed by sympathetic activation, the LS group showed elevated melatonin at 9:30–10:00. This may reflect stress-related chronodisruption, whereby prolonged HPA hyperactivity desynchronizes pineal melatonin release from light-dark cycles. Rodent models daytime melatonin surges under stress, possibly via impaired photic entrainment [41], but this mechanism awaits confirmation in humans.

Consistent with earlier reports [32, 33, 43], HS participants showed higher α-amylase, MT, IgA, lysozyme, and cortisol levels. α -amylase correlated strongly with PSS scores, supporting its relevance as a stress marker. Patients with anorexia nervosa, for example, show altered α-amylase responses to psychosocial stress [32], and portable devices now allow rapid stress assessment [34]. As a marker of sympathetic activity, α-amylase may hold promise for stress research [46]. Cortisol was also elevated in the HS group and has been linked to reduced negative affect [47], suggesting a potential emotional buffering function [40,48,49]. Changes in MT, IgA, and lysozyme may reflect stress-related modulation of circadian rhythms and immunity: elevated melatonin supports sleep-wake regulation [50], [51], increased IgA aids mucosal defense [52], and higher lysozyme enhances antimicrobial capacity [53]. Together, these elevations may represent an adaptive physiological response to stress. Although these biomarkers show promise for stress assessment and monitoring, further validation across diverse populations is needed [54].

α-amylase showed statistically significant but very weak positive correlations with IgA, cortisol, and MT, whereas a strong correlation was observed with lysozyme. MT was weakly positively correlated with IgA and cortisol, and weakly negatively correlated with lysozyme, consistent with prior findings. Given the large sample size, these weak correlations should be interpreted cautiously and not overemphasized as biologically strong relationships [33]. These findings are broadly consistent with previous reports: Rapson et al. also observed positive correlations among α-amylase, IgA, and cortisol [51], [55]. α-amylase and cortisol share similar diurnal fluctuations [52]; and α-amylase activity correlates with norepinephrine release, supporting its use as a non-invasive marker of sympathetic activation [56]. Changes in α-amylase and melatonin have been linked to stress and immune regulation—for example, exercise-induced α-amylase changes correlate with immune parameters [57,58], and melatonin modulates immune function [55,59]. The positive correlation between melatonin and IgA may reflect enhanced immune defense through modulation of immune cells or IgA synthesis [60,61], while its positive correlation with cortisol suggests involvement in HPA axis regulation [62,63]. The negative correlation between melatonin and lysozyme implies a complex immunomodulatory role, possibly via inhibition of lysozyme synthesis or secretion [64]. Collectively, these multi-biomarker correlations offer insights into stress-related physiological and immune processes and support the value of a combined analytical approach.

Diurnal comparisons between HS and LS groups revealed several differences. The HS group showed a higher morning α-amylase peak and a numerically blunted nighttime decline, although this difference was not statistically significant. The overall pattern may still suggest a tendency toward sustained sympathetic activation that warrants further investigation [65], possibly reflecting a shift toward sympathetic dominance that inhibits parasympathetic function [66–68]. This autonomic imbalance may contribute to the observed biomarker patterns [69] and has been associated with increased morning blood pressure and cardiovascular risk [70–72]. Nocturnal lysozyme fluctuations were attenuated in HS individuals, indicating reduced immune rhythmicity. Nighttime cortisol was lower in the HS group [45,73], consistent with HPA axis dysregulation and possibly an adaptive mechanism [74,75]. Melatonin circadian patterns did not differ between groups [76–78], likely reflecting its primary regulation by photoperiod via the SCN [79] and relative insensitivity to acute stress [80], with possible compensatory preservation of sleep cycles [81,82]. IgA levels were elevated in HS at 9:00 AM followed by a sharp decline [83], suggestive of short-term immune activation followed by depletion [84]. CHGA diurnal profiles also differed between groups; its release upon sympathetic activation [85] may be more pronounced in the morning [86]. These diurnal biomarker patterns may offer insights into stress-coping mechanisms and inform individualized stress management approaches [87].

The directionality of immune biomarker changes was unexpected. Contrary to the initial hypothesis that chronic stress would suppress IgA and lysozyme, we observed elevated levels of both in the HS group. This discrepancy could reflect several factors. First, stress in this student sample may be intermittent or acute-on-chronic, potentially activating mucosal immunity via SAM axis stimulation rather than producing the sustained glucocorticoid-mediated suppression typical of chronic stress. Second, the α-amylase-lysozyme correlation (r = 0.52) suggests coordinated neuro-immune activation, possibly reflecting an adaptive phase preceding immune depletion. Third, the 3 PM saliva collection time and the young, healthy sample may capture a distinct phase of stress-related immune modulation. These findings underscore the complexity of stress-immune interactions and suggest that immune activation may characterize certain stress states in otherwise healthy young adults. Longitudinal studies are needed to clarify whether elevated IgA and lysozyme represent an adaptive response preceding immune dysregulation, or a distinct phenotype separate from classical stress-induced immunosuppression.

Fatigue severity and sleep quality emerged as key risk factors for perceived stress in this sample. Interventions targeting these factors could help enhance students’ resilience and mental health. The interplay between fatigue, sleep, and stress has important health implications: poor sleep quality increases fatigue, which in turn worsens stress, creating a self-reinforcing cycle [88–90]. Thus, strategies to improve sleep quality and alleviate fatigue may be central to effective stress management in college populations [91].

Various interventions have been evaluated for reducing stress and improving sleep in college students. Psychological interventions yield moderate-to-large effects on sleep quality (g = 0.61) [92]. Environmental modifications, such as reducing noise disturbances, may also improve sleep and reduce anxiety [93]. Technology-based programs (e.g., STEPS-TECH) have shown benefits for sleep duration and nighttime awakenings. Physical activity enhances sleep quality and emotional well-being [94], indirectly reducing negative affect [95]. Music interventions, particularly self-administered formats, improve sleep and reduce anxiety in students, with longer durations associated with greater efficacy [96]. Combined approaches, such as positive self-hypnosis with resistance training, have also shown promise for stress management^93^. Overall, psychological support, environmental adjustments, exercise, music, and mind-body techniques have demonstrated effectiveness in improving sleep and reducing stress among college students. Given the strong interconnections among fatigue, sleep, and stress, multifaceted strategies addressing these domains are likely to be most beneficial for student health, highlighting the importance of managing these factors to promote both physical and mental well-being.

Several limitations should be considered. Self-reported data may introduce bias, and the sample may not fully represent the broader student population, limiting generalizability. Email-based recruitment could have introduced self-selection bias, as participation required active email engagement. The cross-sectional design precludes causal inferences, and the short study duration limits assessment of long-term effects. Circadian preference was not assessed; given the established links between eveningness, poor sleep, and stress reactivity, the observed biomarker-sleep relationships may be partly influenced by chronobiological differences. These limitations highlight the need for longitudinal studies and clinical validation.

This study highlights the associations among perceived stress, fatigue, and sleep quality in undergraduates, and their collective relevance to mental health. Given the cross-sectional design, the findings suggest that salivary α-amylase, particularly when considered together with self-reported fatigue, may be a promising candidate for further investigation as a stress-related biomarker in future longitudinal studies. While longitudinal validation is needed, these findings may help inform future research on support strategies addressing both psychological and physiological aspects of student stress. Despite its limitations, this study provides a preliminary framework for prospective cohort studies examining stress trajectories.

## Supporting information

S1 Table Sociodemographic of participants. (DOCX)

S2 Table FSS, PSS, and PSQI scores comparisons among the grades. (DOCX)

S3 Table Pearson correlation of PSQI, PSS, and FSS. (DOCX)

S4 Table Pearson correlation of alpha-amylase, IgA, CHGA, MT, LZM, and cortisol. (DOCX)

S5 Table Basic demographic information of participants recruited for diurnal sample collecting. (DOCX)

S1 Fig Scatter plots of MT, CHGA, IgA, LZM, and cortisol versus alpha‑amylase. (TIF)

S1 Text STROBE Statement—Checklist of items that should be included in reports of cross-sectional studies. (DOCX)
