# Supplementary material for: The hidden burden of college life: Stress, sleep, and salivary biomarkers in students—A cross-sectional study
Source: PLOS Ment Health. 2026 Sep 15;3(9):e0000717. doi: 10.1371/journal.pmen.0000717 (PMC13577347; doi:10.1371/journal.pmen.0000717)
Supplement: Supplementary file 1 [file pmen.0000717.s001.docx]

**S1 Table. Sociodemographic of participants.**

|  | **Total (N)** | **Freshmen (n, %)** | **Sophomore (n, %)** | **Junior (n, %)** | **Senior (n, %)** |
| --- | --- | --- | --- | --- | --- |
|  | 637 | 243, 24.3% | 154, 24.20% | 134, 21.00% | 106, 16.60% |
|  | **M ± SD** | **M ± SD** | **M ± SD** | **M ± SD** | **M ± SD** |
| Age | 19.91(1.47) | 18.57(0.81) | 19.88(0.96) | 20.86(0.75) | 21.83(0.72) |
|  | **n(%)** | **n(%)** | **n(%)** | **n(%)** | **n(%)** |
| Gender |  |  |  |  |  |
| *Male* | 217, 34.1% | 66, 27.2% | 51, 33.1% | 60, 44.8% | 40, 37.7% |
| *Female* | 420, 65.9% | 177, 72.8% | 103, 66.9% | 74, 55.2% | 66, 62.3% |
| College |  |  |  |  |  |
| *CAD*^1^ | 57, 8.9% | 15, 6.2% | 11, 7.1% | 12, 9.0% | 19, 17.9% |
| *CST*^2^ | 126, 19.8% | 51, 21.0% | 39, 25.3% | 24, 17.9% | 12, 11.3% |
| *CLA*^3^ | 149, 23.4% | 43, 17.7% | 48, 31.2% | 41, 30.6% | 17, 16.1% |
| *CBPM*^4^ | 305, 47.9% | 134, 55.1% | 56, 36.4% | 57, 42.5% | 58, 54.7% |
| Psychotropic drug use | 23, 3.6% | 6, 2.4% | 3, 1.9% | 4, 3.0% | 1, 0.9% |
| Single-parent family | 52, 8.2% | 20, 8.2% | 19, 12.3% | 9, 6.7% | 4, 3.8% |
| Traumatic events^5^ | 174, 27.3% | 62, 25.5% | 46, 29.9% | 34, 25.4% | 32, 30.2% |
| Affected by COVID-19 severely | 120, 18.8% | 26, 10.7% | 38, 24.7% | 28, 20.9% | 28, 26.4% |

^1^ College of Architecture & Design; ^2^ College of Science, Mathematics and Technology; ^3^ College of Liberal Arts; ^4^ College of Business & Public Management; ^5^ Traumatic events in the last six months include Emotional frustration (e.g., being crossed in love, breaking up with a best friend, death of a close relative)
