# Supplementary material for: The hidden burden of college life: Stress, sleep, and salivary biomarkers in students—A cross-sectional study
Source: PLOS Ment Health. 2026 Sep 15;3(9):e0000717. doi: 10.1371/journal.pmen.0000717 (PMC13577347; doi:10.1371/journal.pmen.0000717)
Supplement: Supplementary file 2 [file pmen.0000717.s002.docx]

**S2 Table. FSS, PSS, and PSQI scores comparisons among the grades.**

|  |  |  |  | mean difference, I-J, *p* | | | |
| --- | --- | --- | --- | --- | --- | --- | --- |
| Scale | Grade |  | Mean score ± SD | Freshmen | Junior | Sophomore | Senior |
| FSS | Freshmen |  | 4.62 ± 0.07 |  | **-0.36, <0.01** | **-0.39, <0.01** | **-0.48, <0.001** |
|  | Junior |  | 4.98 ± 0.08 |  |  | -0.03, 0.81 | -0.12, 0.36 |
|  | Sophomore |  | 5.01 ± 0.09 |  |  |  | -0.09, 0.50 |
|  | Senior |  | 5.10 ± 0.10 |  |  |  |  |
| PSS | Freshmen |  | 38.49 ± 0.48 |  | -0.75, 0.33 | **-2.03, <0.05** | -0.53, 0.54 |
|  | Junior |  | 39.23 ± 0.60 |  |  | -1.28, 0.15 | 0.21, 0.82 |
|  | Sophomore |  | 40.52 ± 0.65 |  |  |  | 1.50, 0.13 |
|  | Senior |  | 39.02 ± 0.73 |  |  |  |  |
| PSQI total | Freshmen |  | 5.41 ± 0.17 |  | -0.23, 0.41 | **-0.88, <0.01** | -0.43, 0.17 |
|  | Junior |  | 5.64 ± 0.66 |  |  | **-0.65,** **<0.05** | -0.20, 0.56 |
|  | Sophomore |  | 6.29 ± 0.23 |  |  |  | 0.45, 0.20 |
|  | Senior |  | 5.84 ± 0.26 |  |  |  |  |
| factor1 | Freshmen |  | 0.96 ± 0.04 |  | -0.01, 0.88 | **-0.22, <0.01** | -0.13, 0.10 |
|  | Junior |  | 0.97 ± 0.05 |  |  | **-0.21, <0.05** | -0.12, 0.17 |
|  | Sophomore |  | 1.18 ± 0.06 |  |  |  | 0.08, 0.35 |
|  | Senior |  | 1.09 ± 0.07 |  |  |  |  |
| factor2 | Freshmen |  | 1.00 ± 0.06 |  | -0.18, 0.06 | **-0.28, <0.01** | **-0.32, <0.01** |
|  | Junior |  | 1.19 ± 0.08 |  |  | -0.10, 0.39 | -0.13, 0.26 |
|  | Sophomore |  | 1.28 ± 0.08 |  |  |  | -0.04, 0.76 |
|  | Senior |  | 1.32 ± 0.09 |  |  |  |  |
| factor3 | Freshmen |  | 0.28 ± 0.03 |  | 0.09, 0.09 | 0.04, 0.44 | 0.11, 0.09 |
|  | Junior |  | 0.18 ± 0.04 |  |  | -0.05, 0.44 | 0.01, 0.86 |
|  | Sophomore |  | 0.23 ± 0.05 |  |  |  | 0.06, 0.38 |
|  | Senior |  | 0.17 ± 0.05 |  |  |  |  |
| factor4 | Freshmen |  | 0.26 ± 0.04 |  | -0.05, 0.44 | -0.07, 0.28 | -0.12, 0.09 |
|  | Junior |  | 0.31 ± 0.05 |  |  | -0.02, 0.75 | -0.72, 0.36 |
|  | Sophomore |  | 0.33 ± 0.05 |  |  |  | -0.05, 0.58 |
|  | Senior |  | 0.38 ± 0.06 |  |  |  |  |
| factor5 | Freshmen |  | 1.05 ± 0.03 |  | -0.08, 0.07 | -0.06, 0.22 | -0.04, 0.45 |
|  | Junior |  | 1.13 ± 0.04 |  |  | 0.03, 0.63 | 0.05, 0.43 |
|  | Sophomore |  | 1.10 ± 0.04 |  |  |  | 0.02, 0.74 |
|  | Senior |  | 1.09 ± 0.04 |  |  |  |  |
| factor6 | Freshmen |  | 0.13 ± 0.03 |  | -0.00, 0.93 | -0.00, 0.96 | -0.00, 1.00 |
|  | Junior |  | 0.14 ± 0.04 |  |  | 0.00, 0.97 | 0.00, 0.95 |
|  | Sophomore |  | 0.13 ± 0.05 |  |  |  | 0.00, 0.97 |
|  | Senior |  | 0.13 ± 0.05 |  |  |  |  |
| factor7 | Freshmen |  | 1.74 ± 0.06 |  | 0.00, 0.92 | **-0.29, <0.01** | 0.08, 0.47 |
|  | Junior |  | 1.73 ± 0.07 |  |  | **-0.30, <0.01** | 0.07, 0.56 |
|  | Sophomore |  | 2.03 ± 0.08 |  |  |  | **0.37, <0.01** |
|  | Senior |  | 1.66 ± 0.09 |  |  |  |  |

Factor1: sleep quality; factor2: sleep latency; factor3: sleep duration; factor4: sleep efficacy; factor5: sleep disturbances; factor6: use of sleep medicine; factor7: daytime dysfunction. *P* values for signiﬁcant comparisons are shown in bold. Total students, N=637； freshmen, n=243; junior, n=154; sophomore, n=134; senior, n=106.
