# Supplementary material for: The hidden burden of college life: Stress, sleep, and salivary biomarkers in students—A cross-sectional study
Source: PLOS Ment Health. 2026 Sep 15;3(9):e0000717. doi: 10.1371/journal.pmen.0000717 (PMC13577347; doi:10.1371/journal.pmen.0000717)
Supplement: Supplementary file 3 [file pmen.0000717.s003.docx]

**S3 Table. Pearson correlation of PSQI, PSS, and FSS.**

|  |  | Pearson correlation, *p* | | |
| --- | --- | --- | --- | --- |
|  | Mean ± SD, N | PSS | FSS | PQSI |
| PSS | 39.18 ± 7.51, 637 |  | **0.41,** **<0.01** | **0.35, <0.01** |
| FSS | 4.87 ± 1.05, 637 |  |  | **0.37, <0.01** |
| PQSI | 5.61 ± 2.71, 637 |  |  |  |

P values for signiﬁcant relations are shown in bold.
