# Supplementary material for: The hidden burden of college life: Stress, sleep, and salivary biomarkers in students—A cross-sectional study
Source: PLOS Ment Health. 2026 Sep 15;3(9):e0000717. doi: 10.1371/journal.pmen.0000717 (PMC13577347; doi:10.1371/journal.pmen.0000717)
Supplement: Supplementary file 4 [file pmen.0000717.s004.docx]

**S4 Tables. Pearson correlation of alpha-amylase, IgA, CHGA, MT, LZM, and cortisol.**

|  |  | Pearson correlation, *p* | | | | |
| --- | --- | --- | --- | --- | --- | --- |
|  | Mean ± SD, N | IgA | CHGA | MT | LZM | Cortisol |
| Alpha-amylase | 13138.66 ± 6739.69 (ng/ml), 262 | **0.14, <0.05** | 0.05, 0.47 | **0.14, <0.05** | **0.517, <0.001** | **0.14, <0.05** |
| IgA | 98385.62 ± 75996.64 (ng/ml), 262 |  | 0.10, 0.09 | **0.29, <0.001** | -0.03, 0.63 | 0.11, 0.08 |
| CHGA | 249.68 ± 305.75 (pg/ml), 262 |  |  | -0.02, 0.70 | -0.08, 0.18 | 0.10, 0.09 |
| MT | 2365.99 ± 3580.72 (pg/ml), 262 |  |  |  | **-0.18, <0.01** | **0.20, <0.01** |
| LZM | 9076.54 ± 5773.76 (ng/ml), 262 |  |  |  |  | -0.09, 0.15 |
| Cortisol | 20.47 ± 32.44 (ng/ml), 262 |  |  |  |  |  |

*P* values for signiﬁcant relations are shown in bold.
