# Supplementary material for: The hidden burden of college life: Stress, sleep, and salivary biomarkers in students—A cross-sectional study
Source: PLOS Ment Health. 2026 Sep 15;3(9):e0000717. doi: 10.1371/journal.pmen.0000717 (PMC13577347; doi:10.1371/journal.pmen.0000717)
Supplement: Supplementary file 5 [file pmen.0000717.s005.docx]

| **S5 Table. Basic demographic information of participants recruited for diurnal sample collecting.** | | | | | | | | | | | | |  | |  |  |
| --- | --- | --- | --- | --- | --- | --- | --- | --- | --- | --- | --- | --- | --- | --- | --- | --- |
|  |  |  |  |  | the night before Sample collecting | | | |  | the morning before 1st Sample collecting | | | | | | |
| ID | sex | weight (kg) | hight (cm) | menstrual cycle | temperature (℃) | blood pressure (mmHg) | blood oxygen saturation% | heart rate (beat/min) | bed time | temperature (℃) | blood pressure (mmHg) | Blood  Oxygen  saturation% | | heart rate (beat/min) | | waken-up time |
| M22502 | male | 69.1 | 171 | / | 36.4 | 103/69 | 98 | 81 | 23:50 | 36.4 | 117/83 | | 97 | 71 | | 8:20 |
| M22503 | male | 54.2 | 172 | / | 36.7 | 116/75 | 99 | 66 | 0:00 | 36.4 | 114/85 | | 99 | 69 | | 8:20 |
| M22504 | male | 50.1 | 176 | / | 36.8 | 115/81 | 99 | 87 | 0:00 | 36.5 | 90/65 | | 98 | 64 | | 8:20 |
| M22505 | male | 63.2 | 168 | / | 36.7 | 120/90 | 98 | 68 | 0:30 | 36.4 | 107/68 | | 99 | 66 | | 8:20 |
| M22506 | male | 44.5 | 163 | / | 36.4 | 90/60 | 98 | 61 | 0:10 | 36.6 | 89/63 | | 99 | 64 | | 8:20 |
| M22507 | male | 53.8 | 170 | / | 36.5 | 112/81 | 98 | 66 | 23:30 | 36.4 | 105/90 | | 98 | 68 | | 8:20 |
| M22508 | male | 65.1 | 171 | / | 36.5 | 100/70 | 97 | 76 | 23:00 | 36.4 | 100/70 | | 98 | 80 | | 8:20 |
| M30307 | male | 45.3 | 167 | / | 36.5 | 95/64 | 98 | 60 | 23:15 | 36.4 | 112/73 | | 99 | 66 | | 8:20 |
| M30308 | male | 75.2 | 183 | / | 36.6 | 100/76 | 98 | 80 | 0:10 | 36.4 | 105/81 | | 98 | 72 | | 8:20 |
| M30309 | male | 84.3 | 178 | / | 36.7 | 102/73 | 98 | 78 | 23:30 | 36.4 | 100/70 | | 99 | 61 | | 8:20 |
| M30310 | male | 90.1 | 181 | / | 36.7 | 120/90 | 97 | 74 | 0:00 | 36.4 | 92/63 | | 97 | 60 | | 8:20 |
| M30311 | male | 80.2 | 181 | / | 36.6 | 117/88 | 97 | 95 | 23:00 | 36.5 | 114/83 | | 98 | 73 | | 8:20 |
| M30312 | male | 75.1 | 176 | / | 36.4 | 110/90 | 98 | 89 | 0:00 | 36.4 | 100/88 | | 98 | 86 | | 8:20 |
| M30313 | male | 65.4 | 178 | / | 36.4 | 100/80 | 98 | 78 | 23:20 | 36.4 | 95/78 | | 99 | 78 | | 8:20 |
| F31712 | female | 55 | 161 | no | 36.7 | 102/72 | 99 | 61 | 0:30 | 36.4 | 104/72 | | 98 | 66 | | 8:20 |
| F31713 | female | 45 | 155 | no | 36.6 | 90/60 | 98 | 71 | 0:00 | 36.4 | 90/60 | | 98 | 68 | | 8:20 |
| F31714 | female | 60 | 165 | no | 36.5 | 95/70 | 99 | 65 | 0:45 | 36.5 | 98/75 | | 98 | 65 | | 8:20 |
| F31715 | female | 52.3 | 162 | no | 36.5 | 90/65 | 98 | 76 | 23:30 | 36.4 | 90/65 | | 99 | 74 | | 8:20 |
| F31716 | female | 49.5 | 165 | no | 36.5 | 90/60 | 98 | 90 | 23:30 | 36.4 | 90/60 | | 97 | 85 | | 8:20 |
| F31717 | female | 60 | 170 | no | 36.5 | 95/65 | 98 | 89 | 23:30 | 36.6 | 95/65 | | 98 | 80 | | 8:20 |
| F31705 | female | 68.0 | 162 | no | 36.6 | 104/78 | 98 | 70 | 0:30 | 36.4 | 100/80 | | 99 | 70 | | 8:20 |
| F31706 | female | 56.1 | 159 | no | 36.6 | 120/90 | 98 | 92 | 23:00 | 36.5 | 107/79 | | 98 | 72 | | 8:20 |
| F31707 | female | 60.0 | 166 | no | 36.1 | 106/84 | 99 | 72 | 11:08 | 36.5 | 116/73 | | 98 | 73 | | 8:20 |
| F31708 | female | 48.0 | 158 | no | 36.7 | 91/64 | 99 | 67 | 22:50 | 36.3 | 89/59 | | 99 | 61 | | 8:20 |
| F31709 | female | 65.2 | 170 | no | 36.7 | 103/61 | 98 | 89 | 23:20 | 36.4 | 99/68 | | 99 | 64 | | 8:20 |
| F31710 | female | 53.2 | 166 | no | 36.7 | 102/62 | 97 | 67 | 23:20 | 36.4 | 91/66 | | 97 | 72 | | 8:20 |
| F31711 | female | 55.1 | 165 | no | 36.7 | 111/77 | 99 | 78 | 23:20 | 36.4 | 98/79 | | 98 | 72 | | 8:20 |
| F22501 | female | 50.0 | 150 | no | 36.6 | 97/68 | 99 | 77 | 23:00 | 36.5 | 95/62 | | 99 | 64 | | 8:20 |
| F22502 | female | 46.9 | 168 | no | 36.5 | 95/66 | 99 | 67 | 0:00 | 36.4 | 90/62 | | 98 | 78 | | 8:20 |
| F30303 | female | 54.8 | 170 | no | 36.6 | 100/62 | 98 | 66 | 0:00 | 36.4 | 92/59 | | 97 | 65 | | 8:20 |
| F30304 | female | 53.6 | 172 | no | 36.5 | 91/60 | 99 | 70 | 23:32 | 36.4 | 89/62 | | 98 | 68 | | 8:20 |
| F30305 | female | 45.2 | 158 | no | 36.7 | 95/68 | 98 | 76 | 0:00 | 36.4 | 90/60 | | 99 | 76 | | 8:20 |
| F30306 | female | 50.5 | 160 | no | 36.7 | 100/70 | 99 | 68 | 0:00 | 36.4 | 95/68 | | 99 | 70 | | 8:20 |
