# Supplementary material for: The hidden burden of college life: Stress, sleep, and salivary biomarkers in students—A cross-sectional study
Source: PLOS Ment Health. 2026 Sep 15;3(9):e0000717. doi: 10.1371/journal.pmen.0000717 (PMC13577347; doi:10.1371/journal.pmen.0000717)
Supplement: Supplementary file 7 [file pmen.0000717.s008.docx]

**Replies to Editor**

1. Please provide a detailed online Financial Disclosure statement. This is published with the article. It must therefore be completed in full sentences and contain the exact wording you wish to be published.

Response: Thank you for your comment. We have revised the Financial Disclosure statement accordingly.

The statement now reads as follows:

“This study was supported by the International Collaborative Research Foundation of WKU (Grant No. ICRP2023006 to HX), Wenzhou-Kean University International Faculty Research Support Program (Grant No. IRSPK202201 to HX), Student Partnering with Faculty Research Program (Grant Nos. WKUSPF2023029 to HX, WKUSPF2023030 to HX, and WKUSPF202439 to HX), and Wenzhou City’s Applied Basic Research (Grant No. GK20250047 to HX). The funders had no role in study design, data collection and analysis, decision to publish, or preparation of the manuscript (Page 33-34, Line 745-751).”

2. Please ensure that the funders and grant numbers match between the Financial Disclosure field and the Funding Information tab in your submission form. Note that the funders must be provided in the same order in both places as well.

Response: Thank you for your comment. We have carefully checked the Financial Disclosure field and the Funding Information tab in the submission form to ensure consistency. The funding sources and their grant numbers have been fully harmonized, and they now appear in identical order across both sections, as required (Page 33-34, Line 745-751).

3. In the online submission form, you indicated that "The datasets used and/or analyzed during the current study available from the corresponding author on reasonable request.".

All PLOS journals now require all data underlying the findings described in their manuscript to be freely available to other researchers, either 
a) In a public repository, 
b) Within the manuscript itself, or 
d) Uploaded as supplementary information.

This policy applies to all data except where public deposition would breach compliance with the protocol approved by your research ethics board. If your data cannot be made publicly available for ethical or legal reasons (e.g., public availability would compromise patient privacy), please explain your reasons by return email and your exemption request will be escalated to the editor for approval. Your exemption request will be handled independently and will not hold up the peer review process, but will need to be resolved should your manuscript be accepted for publication. One of the Editorial team will then be in touch if there are any issues.

For further assistance, you may go to: <http://journals.plos.org/mentalhealth/s/data-availability>

Response: Thank you for your reminder comment. We have contacted the Editorial team, and our exemption request has been approved. The data contain potentially identifiable information from a specific participant group, and participants did not consent to public data sharing. All data access requests will be reviewed and approved by the ethics committee to ensure strict adherence to participant consent and privacy protections. This ensures that data sharing remains possible for legitimate scientific purposes while maintaining appropriate institutional oversight.

**Contact for data access:**
WKU Ethics Committee, Wenzhou-Kean University,  [orspdept@wku.edu.cn](mailto:orspdept@wku.edu.cn)
The corresponding author, Hongyu Xu, [xuhy198169@163.com](mailto:xuhy198169@163.com); [hxu@kean.edu](mailto:hxu@kean.edu)(Page 34-35, Line 762-770).

4. Please provide separate main figure files in .tif or .eps format only and ensure that all files are under our size limit of 10MB.

Response: Thank you for your suggestion. All the figures are uploaded as required.

5. We have noticed that you have uploaded Supporting Information files, but you have not included a list of legends. Please add a full list of legends for your Supporting Information files before or after the references list.

Response: Thank you for your comment. A full list of legends for the Supporting Information files has been added after the list of references.

**Supporting information captions**

**S1 Table**. Sociodemographic of participants.

**S2 Table**. FSS, PSS, and PSQI scores comparisons among the grades.

**S3 Table**. Pearson correlation of PSQI, PSS, and FSS.

**S4 Table.** Pearson correlation of alpha-amylase, IgA, CHGA, MT, LZM, and cortisol.

**S5 Table.** Basic demographic information of participants recruited for diurnal sample collecting.

**S6 Fig.** Scatter plots of MT, CHGA, IgA, LZM, and cortisol versus alpha‑amylase.

**S7 Text.** STROBE Statement—Checklist of items that should be included in reports of cross-sectional studies (Page 48, Line 1052-1061).

6. Some material included in your submission may be copyrighted. According to PLOS’s copyright policy, authors who use figures or other material (e.g., graphics, clipart, maps) from another author or copyright holder must demonstrate or obtain permission to publish this material under the Creative Commons Attribution 4.0 International (CC BY 4.0) License used by PLOS journals. Please closely review the details of PLOS’s copyright requirements here: PLOS Licenses and Copyright. If you need to request permissions from a copyright holder, you may use PLOS's Copyright Content Permission form.

Please respond directly to this email or email the journal office and provide any known details concerning your material's license terms and permissions required for reuse, even if you have not yet obtained copyright permissions or are unsure of your material's copyright compatibility.

Potential Copyright Issues:

Figure 1: Please confirm whether you drew the images / clip-art within the figure panels by hand. If you did not draw the images, please provide (a) a link to the source of the images or icons and their license / terms of use; or (b) written permission from the copyright holder to publish the images or icons under our CC-BY 4.0 license. Alternatively, you may replace the images with open source alternatives. See these open source resources you may use to replace images / clip-art:
- https://commons.wikimedia.org
- <https://openclipart.org/>

Response: Thank you for your comment. Figure 1 was created using BioRender. The figure is original and all elements were hand-drawn by the authors using BioRender’s illustration tools. The figure is published under the Creative Commons Attribution 4.0 International (CC BY 4.0) license in compliance with PLOS’s copyright policy. The BioRender-generated figure can be accessed at: https://BioRender.com/i61e911 (Wong, A., 2026). We confirm that no third-party copyrighted material is included in this figure (Page 9, Line 196-197).

7. If the reviewer comments include a recommendation to cite specific previously published works, please review and evaluate these publications to determine whether they are relevant and should be cited. There is no requirement to cite these works unless the editor has indicated otherwise.

Response: Thank you for your reminder.

8. Please review your reference list to ensure that it is complete and correct. If you have cited papers that have been retracted, please include the rationale for doing so in the manuscript text, or remove these references and replace them with relevant current references. Any changes to the reference list should be mentioned in the rebuttal letter that accompanies your revised manuscript. If you need to cite a retracted article, indicate the article’s retracted status in the References list and also include a citation and full reference for the retraction notice.

Response: Thank you for your comment. We have carefully reviewed the list of references to ensure completeness and correctness. No retracted articles were cited. The list of references has been restructured according to the journal’s style: author punctuation has been corrected, and publication details have been reorganized into the correct chronological format. All changes applied to the list of references are detailed in the rebuttal letter (Page 36-48, Line 774-1050).

**Replies to Reviewer 1**

**Reviewer #1: General information**

1. The journal requires double spacing.

**Response**: Thank you for your comment. The manuscript has been entirely reformatted to double spacing, as required by the journal.

2. I recommend that authors use the same font and font size for the main text and the bibliography (main text: Times 12/bibliography: Calibri 14).

**Response**: Thank you for your comment. The main text and the list of reference have been standardized to Times New Roman, 12 pt, ensuring consistent font and size throughout the manuscript.

3. To comply with the journal’s submission guidelines, the References section will need to be reformatted, mainly to adjust the authors’ punctuation and to organize the publication details in the correct chronological format.

**Response**: Thank you for your comment. The list of references has been reformatted according to the journal’s guidelines. Author punctuation has been corrected, and publication details have been reorganized into the correct chronological order (Page 36-48, Line 774-1050).

4. Materials and Methods? Instead of methods

**Response**: Thank you for your comment. The section originally entitled “Methods” has been renamed to “Materials and Methods” to align with the journal’s conventions (Page 7, Line 155).

**Ending section**

5. The following elements are required, in order:

Acknowledgments

References

Supporting information captions (if applicable)

**Response**: Thank you for your comment. The concluding sections of the manuscript have been accordingly reordered as required: Acknowledgments, followed by References, and then Supporting Information captions (where applicable) (Page 33-48, Line 744-1061).

6. Figure and table placement

**Response**: Thank you for your comment. Figure captions have been placed immediately after the first paragraph where each figure was cited, and tables were inserted immediately after the first paragraph where each table was cited. All figure files and supporting information files have been prepared for separate upload accordingly.

7. Add short title no longer than 70 characters

**Response**: Thank you for your comment. A short title (no more than 70 characters) has been added to the title page: Stress, Sleep, Fatigue, and Salivary Biomarkers in College Students (68 characters) (Page 1, Line 4).

8. Fix bibliography format. Correct example Published articles

**Response**: Thank you for your comment. The list of references has been reformatted to comply with the journal’s requirements. Author punctuation has been corrected, and publication details were organized in the correct chronological order, as illustrated in the provided example (Page 36-48, Line 774-1050).

**Abstract**

1. The abstract states that “90.4% experienced clinically significant stress.” However, when reviewing the results in the body of the manuscript, that 90.4% is broken down into ‘moderate’ (56.83%) and “high” (33.59%) stress. Labeling overall moderate stress as “clinically significant” in the abstract could be considered an overly strong or alarmist statement that does not accurately reflect the categorization of the PSS-14 scale detailed in the methods.

**Response**: Thank you for your comment. We agree with your concern. The original phrasing “clinically significant stress” was imprecise and might overstate the findings. To align with the Results section and the PSS-14 categorization described in the Methods, we have revised the Abstract as follows: “90.4% experienced moderate-to-high perceived stress.” This change accurately reflects the data and avoids any unintended overinterpretation (Page 2, Line 27-28).

2. The abstract indicates HPA axis hyperactivity by stating “cortisol↑.” While useful for summarizing, the main text details that students with high stress show an atypical diurnal pattern (lack of normal morning peak and an anomalous peak at 2:00 p.m., along with elevated nighttime levels). A simple “cortisol↑” misses the nuance that the actual finding is an alteration in the diurnal rhythm rather than a simple basal increase.

**Response**: Thank you for your comment. We appreciate this important finding. The original notation “cortisol↑” oversimplified the diurnal dysregulation found in the high-stress group. To more accurately reflect the findings in the main text, we have revised the Abstract as follows: “hypothalamic-pituitary-adrenal (HPA) axis dysregulation is characterized by altered diurnal cortisol patterns.” This revision captures the complexity of the cortisol findings without overgeneralizing (Page 2, Line 31-35).

3. The abstract is very competent and appealing to readers. Is necessary a minor adjustment in the wording about “clinically significant stress” to better align it with the results data.

**Response**: Thanks for your comment. As detailed above, we have revised the wording to better align with the results data. The Abstract has been accordingly revised as follows: “revealing that 90.4% experienced moderate-to-high perceived stress.” This adjustment ensures consistency with the manuscript’s Results and Methods sections while maintaining the Abstract’s clarity and appeal (Page 2, Line 27-28).

**Introduction**

1. Lines 57-60: I suggest that the authors ensure that all theoretical statements in the “Introduction” section are properly supported with their respective bibliographic references.

**Response**: Thanks for your comment. We have carefully reviewed the Introduction and added the necessary bibliographic references to support all theoretical statements. Specifically, we have supplemented citations where foundational concepts or mechanistic claims were previously under-referenced. These additions are marked in the revised manuscript (Page 5, Line 93-95).

2. Lines 76-112: This paragraph is very long (it occupies almost 36 continuous lines in the original manuscript). The density of medical acronyms and signaling cascades causes cognitive fatigue in the reader. I recommend that the authors strategically divide the paragraph or summarize the information.

**Response**: Thanks for your comment. This paragraph has been strategically divided into two shorter paragraphs to improve readability. The content has also been concisely summarized, reducing the density of acronyms and signaling cascades while preserving key mechanistic details. The revised structure enhances clarity and reader comprehension(Page 6-7, Line 110-138).
3. Lines 125-130: The final results of the study should not be revealed in the Introduction. This section is for stating the state of the art of the study idea.

**Response**: Thanks for your comment. The sentences that prematurely reported study findings have been removed from the Introduction. This section currently appropriately concentrates on the rationale, gaps in the literature, and the study’s objectives without revealing specific results (Page 7, Line 139-153).

4. Lines 123-125: The paragraph states that the study proposes a “novel self-assessment paradigm” that allows for non-invasive quantification of physiology. The actual methodology of the study requires participants to donate 11 saliva samples over 24 hours under the supervision of technicians and in designated rooms. This is a rigorous experimental design, not a simple self-assessment. It is recommended to change the term to “novel non-invasive psychobiological profiling approach” or similar.

**Response**: Thanks for your comment. The phrase “novel self-assessment paradigm” has been replaced with “novel non-invasive psychobiological profiling approach” to more accurately reflect the rigorous experimental design involving supervised diurnal saliva collection. This revision aligns the description with the actual methodology presented in the Materials and Methods section (Page 6, Line 121-123).

5. Lines 139-150: Methodologically, it is not correct to describe the study in this section. I recommend that the authors delete this part or move it to the discussion section.

**Response**: Thanks for your comment. The methodological details originally present in this section have been deleted from the Introduction to maintain the section’s concentration on background, rationale, and objectives. This ensures adherence to the standard scientific writing (Page 7, Line 139-153).

6. In the last paragraph of the introduction, the text hypothesizes “IgA/LZM suppression.” However, the abstract and results of the article reveal that what was actually found was an activation/elevation of immunity in highly stressed students (IgA↑, lysozyme↑). I suggest revising the “Discussion” section to explicitly and clearly address why the authors expected immune suppression but ended up finding activation.

**Response**: Thanks for your comment. We have added a dedicated paragraph to explicitly address the unexpected finding of elevated IgA and lysozyme levels in high-stress participants. We discussed potential explanations, including the possibility that acute or intermittent stress might transiently enhance mucosal immune activity, and the observed pattern might reflect a state of immune activation rather than suppression, contrasting with the classic chronic stress-induced immunosuppression model. This addition provides a more detailed interpretation of the findings and aligns with the results presented (Page 28-29, Line 627-644).

**Methods**

1. Line 178: I suggest the authors change the verb tense to past tense: “Participants are permitted to maintain their regular eating...”

**Response**: Thanks for your comment. We have revised the verb tense to past tense. The sentence was revised as follows: “Participants were permitted to maintain their regular eating, studying, and exercising routines, in alignment with their typical campus lifestyle (Page 8, Line 166-169).”

2. The text states at the beginning that students were recruited between “March 3, 2023, and May 5, 2023” (a period spanning just over two months). However, a few lines below, the same paragraph concludes by stating that “Data collection occurred over three weeks.” I recommend that the authors define the exact period.

**Response**: Thanks for your comment. To clarify, recruitment spanned the full period from March 3 to May 5, 2023, while the actual questionnaire and sample collection were conducted intensively over three weeks in this period. The following revision was accordingly applied: “Recruitment and questionnaire data collection were conducted over three weeks in this period (Page 8, Line 172-173).”

3. Lines 198-201: What was the exact alpha level (e.g., 0.05)? What was the statistical power achieved (e.g., >80%)? What was the effect size detected?

**Response**: Thanks for your comment. We have added the requested details to the post-hoc power analysis description. The sentence has been revised as follows: “With an alpha level of 0.05 and a sample size of 637 for questionnaire analyses and 33 for diurnal profiling, the statistical power exceeded 80% for detecting medium-to-large effect sizes (Cohen’s d ≥ 0.5) (Page 9, Line 191-193).”

4. Line 196: The authors mention “A random selection of 285 students from the full sample was made for biomarker analysis,” but in the results section they mention “Totally, 262 salivary samples were collected at 3 PM and analyzed using ELISA.” How do the authors explain this difference?

**Response**: Thanks for your comment. Of the 285 randomly selected participants, 23 samples were excluded due to insufficient saliva volume or poor sample quality, resulting in 262 valid samples for ELISA. We have added this clarification to the manuscript: “Of these, 23 samples were excluded due to insufficient volume or poor quality, resulting in 262 samples for final ELISA (Page 9, Line 187-189).”

5. Lines 217-219: I recommend that the authors remove the mention of the FSS and PSQI alphas from the PSS paragraph. Each paragraph should describe only its own assessment tool to maintain a logical reading flow.

**Response**: Thanks for your comment. The references to FSS and PSQI Cronbach’s alpha values have been removed from the PSS paragraph and appeared only in their respective paragraphs.

6. Lines 241-242: I recommend that the authors clarify whether participants had to meet all criteria simultaneously (“and”) or only one of them (“or”).

**Response**: Thanks for your comment. We have clarified the selection criteria for both groups. The following revisions were accordingly applied: “The LS group met the following criteria: PSS ≤ 28, PSQI ≤ 5, and FSS < 4.0 (all the three criteria required simultaneously). The HS group included participants who met at least one of the following criteria: PSS ≥ 57 or PSQI ≥ 5 or FSS ≥ 5 (Page 11, Line 237-238).”

7. Line 241: In the “Instruments and Data Collection” section, good sleep quality is defined as “PSQI ≤ 5.” However, this section states that to enter the Low Stress (LS) group, participants had to have “PSQI < 5.” If a student scores exactly 5 on the PSQI, do they qualify for the LS group or are they excluded? Mathematical notation should be perfectly consistent throughout the manuscript.

**Response**: Thanks for your comment. To ensure consistency with the established cutoff (PSQI ≤ 5 for good sleep quality), we have revised the LS group criterion to “PSQI ≤ 5”. This aligns with the definition in the Instruments section and ensures that participants with a score of exactly 5 are included in the LS group (Page 11, Line 239).

8. Line 253: I recommend that the authors use verbs in the past tense, given that these activities have already been carried out.

**Response**: Thanks for your comment. We have changed the relevant verbs to past tense. The following revisions have been applied: “The encapsulated sample’s EP tube was stored in tin foil to protect it from light, and strict adherence to light protection protocols was maintained throughout the experiment (Page 12, Line 250-251).”

9. Lines 303-306: It is suggested to explicitly indicate which post hoc test was applied after the ANOVA (e.g., Bonferroni or other correction).

**Response**: Thanks for your comment. We have added the post-hoc test information to the Statistical analysis section. The following revisions have been applied: “When ANOVA revealed significant differences, post-hoc comparisons were performed using the least significant difference (LSD) test (Page 14, Line 301-303).”

10. Lines 318-319: I suggest that the authors indicate "if the data were not normal, did they transform them logarithmically before applying ANOVA and Pearson? Or did they use nonparametric alternatives (such as Spearman's correlation or Kruskal-Wallis/Mann-Whitney tests)? I believe this should be clearly specified.

**Response**: Thanks for your comment. We have added the requested clarification to the Statistical analysis section. The following revisions have been applied: “For variables that deviated from normality, Spearman’s rank correlation was used as a non-parametric alternative. When normality assumptions were violated for repeated-measures data, logarithmic transformation was applied prior to analysis (Page 14, Line 298-299).”

**Results**

1. In one paragraph, the authors state that second-year students had significantly higher scores on the PSS compared to first-year students (p = 0.03). However, in the following paragraph, they conclude that “No significant variations in prevalence were identified across academic years (χ²=6.87, p=0.65).” Although mean scores may differ statistically without affecting the overall prevalence categorization, this distinction may not be entirely clear to readers. It would be helpful to include a brief clarifying statement indicating that, while raw mean scores varied (with second-year students showing higher values), the distribution across clinical categories (low, moderate, and high) did not differ significantly.

**Response**: Thanks for your comment. We have added a clarifying sentence at the end of the prevalence paragraph to explicitly address the distinction between mean score differences and categorical prevalence. The following revisions were applied: “Although sophomores exhibited higher mean PSS scores compared with freshmen, the distribution across stress severity categories (low, moderate, high, very high) did not significantly differ by the academic year (Page 17, Line 374-376).”

2. Lines 401-402: The acronym MT is repeated in the title and IgA is omitted, even though it is also analyzed in this section.

**Response**: Thanks for your comment. The section title originally read: “The relationships among concentrations of salivary alpha-amylase, MT, CHGA, LZM, cortisol, and MT,” which contained a duplicate “MT” and omitted “IgA.” We have corrected the title as follows: “The relationships among concentrations of salivary alpha-amylase, MT, CHGA, IgA, LZM, and cortisol.” This correction ensures that all six biomarkers analyzed in this section were accurately listed without duplication (Page 19, Line 424-425).

3. Lines 412.420 I recommend that the authors add the p-values to support the differences.

**Response**: Thanks for your comment. We have added the corresponding p-values to the comparisons between the low alpha-amylase (LA) and high alpha-amylase (HA) groups. The following revisions were applied: “Participants were divided into low alpha-amylase (LA; mean = 8.98 μg/ml) and high alpha-amylase (HA; mean = 20.10 μg/ml) groups using K-means clustering. A two-way ANOVA revealed that the HA group had significantly higher levels of lysozyme (LZM; *p*<0.001; Fig 5E) and cortisol (*p*<0.01; Fig 5F) compared with the LA group. No significant differences were identified for IgA (*p*=0.12; Fig 5B) or melatonin (MT; *p*=0.17; Fig 5D). However, chromogranin A (CHGA) did not follow this pattern (*p*=0.70; Fig 5C). For all biomarkers, no significant group-by-gender interaction effects were detected (all *p*>0.05). Notably, a significantly larger proportion of women than men exhibited elevated cortisol concentrations (*p*<0.01; Fig 5F).” These additions provide the necessary statistical support for the reported differences (Page 20, Line 435-443).

**Discussion**

1. The discussion adequately integrates psychological and biological findings, which helps reinforce the impact of the study. However, a careful final review of the text is recommended, as there are some duplicate paragraphs that should be corrected.

**Response**: Thanks for your comment. We have thoroughly reviewed the Discussion section and removed the duplicate paragraphs as recommended. The revised Discussion is currently more concise and cohesive, involving all key scientific contents (Page 24-33, Line 523-742).

**Replies to Reviewer 2**

**Reviewer #2:**

This article examines the stress in undergrads by measuring many salivary biomarkers as well as questionnaires. This should provide insight into salivary biomarkers. The article merits publication; however, some points should be addressed first.

**Title**

1. I'm not sure if "a cross-sectionaly study" should be added.

**Response**: Thanks for your comment. To enhance clarity and align with common practice in epidemiological and psychobiological research, we have added the study design specification to the title (Page 1, Line 1-2).

**Abstract**

1. It would be nice to also note the p value when reporting r values.

**Response**: Thanks for your comment. To provide complete statistical information and enhance the transparency of the results, we have added the corresponding p-values alongside each reported correlation coefficient to the Abstract (Page 2, Line 29-36).

**Results**

1. The fonts in all the figures are very small and barely intelligible. It would be nice to adjust the make them more readable. For example, the numbers are so tiny and just by not keeping decimal could make it more easily to read.

**Response**: Thanks for your comment. We have carefully revised all figures to improve their readability.

2. Some values are in the range of 100,000 ng/ml. It would be more easily understandable if using ug/ml so the numbers are smaller amount.

**Response**: Thanks for your comment. In the main text, we have converted the concentration units of salivary alpha-amylase from ng/ml to μg/ml to improve readability. (Page 20, Line 435-436) However, in the heatmaps (Fig 3), Boxplots (Fig 5) and the scatter plots (S6 Fig), the original units (ng/ml or pg/ml) have been retained. This is because these figures directly present the raw data as measured by the ELISA kits, whose standard output units are ng/ml or pg/ml. Keeping the original units in the figures ensures consistency with the actual experimental measurements and avoids potential rounding errors or confusion that might arise from unit conversion. If the reviewer strongly prefers uniform units throughout the manuscript, we are ready to convert all figures to μg/ml and update the figure legends accordingly.

3. Fig2: It would be nice to also indicate which range of scores means mild/mod/severe

**Response**: Thanks for your comment. To help readers interpret the distribution of PSS scores more easily, we have added a clarifying statement to the legend of Fig 2.

4. PSQI in Fig 2 use score 7 which is different from the text that use 5. Please check

**Response**: Thanks for your comment. We thank the reviewer for identifying this discrepancy, which has been resolved accordingly (Page 16, Line 345-356).

5. Please check scores for FSS in Fig 2. I think there is some mistake. The % and score don't match with the text.

**Response**: Thanks for your comment. To ensure consistency between the figure and the text, we have added a clarifying statement to the legend of Fig 2, clarifying the FSS score ranges corresponding to moderate and severe fatigue (Page 16, Line 345-356).

6. For all figures, please use the symbol ≤ not <= as it's very difficult to read. Would also be nice to state HS LS groups in the x axes.

**Response**: Thanks for your comment. We have reviewed all figures and captions and made the necessary revisions to enhance their readability and ensure compliance with standard scientific formatting.

7. Fig 3 and 5: It's impossible to tell which color represent what values as they are quite similar. There are 6 colors in the axes while in the figure there are 4. Maybe showing the box-plot like in Fig 2 would be easier to understand. Or should indicate the values in the text. ALso please avoid redundancy, like indicationg IgA (Immunoglobulin A), choose one.

**Response**: We thank the reviewer for the suggestion. We converted Fig 5 to a box plot to better show group comparisons based on alpha-amylase levels. For Fig 3, we retained the heatmap because it presents a two‑dimensional stratification (stress × gender) across multiple biomarkers, and heatmaps compactly display the magnitude of each value. We have improved the legend and caption to make the heatmap more interpretable. We appreciate the reviewer’s understanding (Page 20, Line 435-447). The example figures are attached below:

**Fig 3A	Fig 5A**

8. Fig 2E and 2F: it's a bit surprising that LS and HS groups had different PSQI and FSS since the box plot showed quite similar values. Please check.

**Response**: Thanks for your comment. To improve clarity, we have made the following additions to the manuscript: (1) In the Results section, we have added the mean values and standard deviations for PSQI and FSS scores in each group to provide quantitative context for the statistical significance; (2) In the figure legend, we have included a clarifying statement noting that, despite overlapping distributions, the differences in group means are statistically significant (two-way ANOVA; (3) In the figure itself, we have added significance markers (e.g., ***) to the box plots in Fig 2E and 2F to visually indicate group differences. These revisions help readers correctly interpret the findings. (Page 16, Line 345-356)

9. Pearson's correlations were actually very weak (for example, only r=0.14 for amylase vs IgA). It would be nice to show the scatter plot of these values in supplementary file.

**Response**: Thanks for your comment. To provide readers with a more complete visual representation of the correlation results, we have created a set of scatter plots for all key biomarker correlations reported in the manuscript. These have been compiled as S6 Fig. The example figure is attached below:

eg.

10. It would be nice to indicate the unit of the biomarkers values in figure legends or in the figures themselves.

**Response**: Thanks for your comment. To ensure readers can accurately interpret the data presented in the figures, we have added unit specifications to both the figure legends and, where applicable, the figure axes.

11. Fig 6 should really be adjusted as the current version is intelligible. It's too small. Actually the left and middle panels are not necessary as both are already shown in the right panels.

**Response**: Thanks for your comment. To improve readability, the original Fig 6 has been split into three separate figures (new Figs 6, 7, and 8), each with enlarged fonts and clearer formatting. Regarding the left and middle panels, we respectfully note that they are not redundant, as the y-axis units differ among the panels and each panel conveys distinct experimental results (e.g., raw diurnal profiles for LS and HS groups separately). The right panels present group comparisons based on GEE analysis. Therefore, we have retained all panels, while presented them in a larger, more legible format across multiple figures. We confirm that this revision addresses the reviewer’s concern while preserving the full dataset (Page 21-22, Line 459-479).

**Discussion:**

1. It's very long and tedious to read. Some information is redundant or mentioned many times.

**Response**: Thanks for your comment. The Discussion section has been thoroughly revised to remove redundant information and improve conciseness, while retaining all essential content. We confirm that the revised version is currently clearer and more focused (Page 28-31, Line 661-680).

2. It would also be very nice to make a paragraph shorter for readability.

**Response**: Thanks for your comment. To improve readability, we have restructured several overlong paragraphs in the Discussion section by splitting them into shorter, thematically concentrated units. All key content has been retained, and the revised text now reads more smoothly (Page 28-31, Line 661-680).
