# Supplementary material for: The hidden burden of college life: Stress, sleep, and salivary biomarkers in students—A cross-sectional study
Source: PLOS Ment Health. 2026 Sep 15;3(9):e0000717. doi: 10.1371/journal.pmen.0000717 (PMC13577347; doi:10.1371/journal.pmen.0000717)
Supplement: Supplementary file 9 [file pmen.0000717.s010.doc]

First of all, we would like to express our sincere gratitude to the reviewers for their constructive and positive comments.

**Replies to Reviewer 2**

Minor considerations:

1. SAM is abbreviation. please write full name at first appearance.

**Response**: We thank the reviewer for this reminder. We have expanded "SAM" to "sympathetic-adrenal-medullary (SAM)" at its first appearance in the Introduction. The revised sentence now reads: "Salivary α-amylase is a marker of sympathetic-adrenal-medullary (SAM) axis activation, rising rapidly via β-adrenergic signaling during acute stress..." (Introduction section, p. 4, lines 63–65).

2. Line 166: Participants for diurnal sample collection. From the way it's written: 15 participants met LS criteria, and 18 met HS criteria. It seems strange that from 6xx subjects only 33 met the criteria. The LS is possible because all 3 criteria are required (PSS<28 + PSQI <5 + FSS <4) but for HS group only 1 criteria is required (either PSS >57 or PSQi >5 or FSS >5).

**Response**: We agree with the reviewer that the selection process needed clarification. The initial screening identified 40 participants who met at least one HS criterion; however, 22 of them were excluded due to ongoing psychotropic medication use (e.g., sertraline, trazodone), per our pre-established inclusion criteria. This left 18 participants in the final HS group. We have added the following explanation to the Methods section (see revised manuscript, “Participants for Diurnal Sample Collection” section): “Initially, 40 participants met at least one HS criterion; of these, 22 were excluded due to ongoing psychotropic medication use (e.g., sertraline, trazodone), leaving 18 participants in the final HS group.” ("Participants for Diurnal Sample Collection" section, p. 8-9, lines 170–173).

3. -Line 290: "MT concentrations showed positive correlations with concentrations of correlated positively with IgA." Please correct.

**Response**: We apologize for this typographical error. The sentence has been corrected to: “MT concentrations showed positive correlations with IgA (r = 0.29, p < 0.001) and cortisol (r = 0.20, p < 0.01), and negatively with lysozyme (r = -0.18, p < 0.01)” (Results section, p. 14, lines 293–295).

4. -Mistake in Fig 7D and 7E. the graphs superimposed. Also mistake in the Discussion (line413) "HS group showed elevated melatonin at 9:30". Actually it's LS group, please check.

**Response**: We thank the reviewer for identifying both issues.

(1) Figure 7D and 7E overlay: We have regenerated the figure to resolve the superimposition issue and have uploaded the corrected version as a separate file.

(2) Melatonin group reference: We have corrected the Discussion (line 413) from “the HS group showed elevated melatonin at 9:30-10:00” to “the LS group showed elevated melatonin at 9:30-10:00,” which is consistent with Fig 7D–F (Discussion section, p. 19, lines 416–417).

5. -Line 451: the blunted nighttime decline of amylase was not significantly different between HS and LS group, so cannot really conclude so.

**Response**: We agree with the reviewer's observation. We have revised the Discussion to reflect that the nighttime decline was numerically blunted in the HS group but not statistically significant. The revised text now reads: “The HS group showed a higher morning α-amylase peak and a numerically blunted nighttime decline, although this difference was not statistically significant. The overall pattern may still suggest a tendency toward sustained sympathetic activation that warrants further investigation.” (Discussion section, p. 21, lines 453–457).
