# Supplementary material for: The hidden burden of college life: Stress, sleep, and salivary biomarkers in students—A cross-sectional study
Source: PLOS Ment Health. 2026 Sep 15;3(9):e0000717. doi: 10.1371/journal.pmen.0000717 (PMC13577347; doi:10.1371/journal.pmen.0000717)
Supplement: Supplementary file 8 [file pmen.0000717.s009.docx]

First of all, we would like to express our sincere gratitude to the reviewers for their constructive and positive comments.

**Replies to Reviewer 1**

Reviewer #1: The authors complied with most of the requested guidelines regarding formatting, restructuring and methodological clarifications:
1. General format and structure
The manuscript was fully standardised in terms of formatting, using double spacing and 12-point Times New Roman font throughout all sections, including the references. Furthermore, the formal presentation of the article was enhanced by incorporating the methodological design into the title (“A Cross-Sectional Study”) and creating an appropriate short title of 68 characters. In the methodology section, the heading was correctly amended to “Materials and Methods”, in line with standard scientific nomenclature. Finally, the references were reorganised according to the Vancouver/PLOS format, correcting the chronological order and author punctuation, and removing residual metadata that remained in the document.
**Response:** We sincerely thank the reviewer for the accurate evaluation and for acknowledging the revisions undertaken in response to the previous round of comments. We have carefully re-examined the manuscript to ensure that all remaining inconsistencies have been fully addressed.

Although the manuscript shows significant improvements and is generally of a high standard, there are still some areas that require further revision. In particular, certain corrections mentioned by the authors in their rebuttal letter have not been fully incorporated into the current version of the manuscript, and some methodological justifications could be strengthened to ensure greater clarity and consistency prior to formal acceptance.

1. Critical Discrepancy in PSQI Cut-off Points

A significant inconsistency remains in the definition of poor sleep quality. The methodological text sets the cut-off point as PSQI >5, whereas the caption for Figure 2 uses PSQI >7. In their response letter, the authors explicitly stated that this discrepancy had been corrected (“We thank the reviewer for identifying this discrepancy, which has been resolved accordingly”); however, upon reviewing the current version of the manuscript, I found that the problem persists. On page 16, lines 347–348, the caption for Figure 2 continues to state: “Participants with PSQI >7 were classified as having poor sleep quality”, contradicting the methodology described previously. I consider that this inconsistency must be corrected before acceptance, as it affects the internal coherence of the manuscript and reflects insufficient editorial review of the final version.

2. Inconsistency of Units in the Text vs. Figures.

I previously requested that the units of measurement shown in the figures be changed, replacing values expressed in ng/ml with more readable units, such as g/ml, in order to facilitate the visual interpretation of the data. In their response, the authors state that they made this change only in the text of the manuscript, but decided to retain the original units (“ng/ml” and “pg/ml”) in the heatmaps and boxplots, arguing that these correspond to the raw data obtained directly from the ELISA kits and that this would avoid potential conversion errors.
However, I consider that this justification does not adequately resolve the issue raised. Currently, the manuscript presents values such as “8.98 g/ml” in the text and “8980 ng/ml” in the figures simultaneously, which hinders comparative reading and may cause unnecessary confusion for the reader. From an editorial perspective, it is essential to maintain consistency in the units of measurement throughout the manuscript. I therefore recommend standardising the units used.

**Response:** Thanks for your comment. We apologize for the oversight regarding the two inconsistencies you identified. We have thoroughly corrected them as detailed below.

1. PSQI cutoff point inconsistency (legend of Fig. 2)

We sincerely regret that the correction we previously stated in our rebuttal letter was not fully implemented in the manuscript. The legend of Figure 2 incorrectly stated “PSQI > 7”. This has been corrected as “PSQI > 5” to match the Methods section and the main text. The revised legend is shown below with the change highlighted (p11, lines 233-234).

2. Inconsistency of units between text and figures

To fully resolve the reviewer’s comment, we have standardized all units across the manuscript. Specifically:

(1) Alpha-amylase, IgA, and lysozyme were uniformly presented in μg/ml (both in text and figures).
(2) Cortisol was presented in μg/dl (both in text and figures).
(3) Melatonin (MT) and Chromogranin A (CHGA) were presented in pg/ml.

Corresponding numerical values in Fig. 3, Fig. 5, Fig.6, Fig.7, Fig. 8, and Supplementary Fig. S6 have been recalculated and updated. For example, in Fig. 3, alpha-amylase values originally shown as “20,097.53 ng/ml” were displayed as “20.10 μg/ml”. In Fig. 5, the units presented in the box plots have been standardized to correspond with those reported in the main text. The figure legends have also been revised accordingly to ensure consistency in unit presentation. (p13, lines 277-278; p14, lines 303-306)

**Replies to Reviewer 2**

Reviewer #2: The authors have made effort to improve the manuscript. However, there are some issues of concern.
Actually, some of the issues have not been addressed even though the authors stated so.
-For example, Figure 2. There are still discrepancies between text and figure legend. Nothing changed.
In the text, PSQI was cutoff at 5 (Line 341) while in Figure legend PSQI cutoff was 7 (Line 348).

**Response:** Thanks for your comment. The two issues you raised have been fully corrected in the manuscript.

1. PSQI cutoff inconsistency (legend of Figure 2 vs. text)

We regret that the previous correction was not properly incorporated into the manuscript. In the current version, the legend of Figure 2 incorrectly stated “PSQI > 7.” This has been corrected to “PSQI > 5,” in accordance with the definition provided in the Methods section (line 341). The revised legend is provided below (see revised manuscript, Fig. 2 legend, p11, lines 233-234).

2. Unit inconsistency between text and figures

We have fully standardized units throughout the manuscript. All biomarker concentrations are presented consistently in the text, figures, and figure legends as follows:

| **Biomarker** | **Standardized unit** |
| --- | --- |
| Alpha-amylase | μg/ml |
| IgA | μg/ml |
| Lysozyme | μg/ml |
| Cortisol | μg/dl |
| Melatonin (MT) | pg/ml |
| Chromogranin A (CHGA) | pg/ml |

Corresponding numerical values in Fig. 3, Fig. 5-8, and Supplementary Fig. S6 have been converted accordingly (e.g., 20,097.53 ng/ml → 20.10 μg/ml). Figure legends have been updated to reflect these units. The text already used these standardized units (e.g., “8.98 μg/ml” and “20.10 μg/ml”). There is no longer any discrepancy (see revised manuscript, Fig. 3 legend, Fig. 5 legend, Figs. 6-8 legends, and S6 Fig legend, pp13, lines 277-278; p14, lines 303-306).

-The discrepancies regarding FSS values still remained. 51.02% = mod fatigue, 31.55%= severe fatigue (Line343-344). But in the Fig2C, FSS >5 is 51.02% and FSS 4-4.9 =31.55%.

**Response:** Thanks for your comment. Upon reviewing the original data, we confirmed that the percentages shown in the figure are correct: FSS ≥5, indicating severe fatigue, was 51.02%, and FSS 4-4.9, indicating moderate fatigue, was 31.55%. The corresponding values in the text had been inadvertently reversed. We have corrected the text accordingly (see revised manuscript, Results section, p11, lines 228-229).

-Fig 2 D-F showed graph as male female while the text explain difference between LS and HS groups withour differentiang sexes. The significant difference maker was on either male of female in the Fig which is misleading. Should clearly state significant difference compare with what group.

**Response:** Thanks for your comment. The two-way ANOVA indicated a significant main effect of stress group (LS vs. HS) on both PSQI and FSS scores, with no significant group-by-gender interaction (p > 0.05). Therefore, the difference between LS and HS groups was consistent across both male and female genders. To avoid misleading readers, we have made the following revisions:

(1) Revised figure: Figures 2D-F have been revised to display only two groups (LS and HS), with male and female data combined, as the statistical analysis did not indicate a significant interaction effect. Significance markers have been adjusted accordingly to directly denote the differences between the LS and HS groups.
(2) Updated figure legend: The legend currently states clearly that the significance markers refer to the comparison between LS and HS groups (main effect). The original gender-specific panels have been replaced with a single box plot per stress group (see Fig 2D, E & F).
(3) Text clarification: In the Results section, we have explicitly stated that the LS-HS difference was significant for both PSQI and FSS (p < 0.001), and no significant gender-based differences or interactions were found (p11,12, lines 234-241).

-This situation of sex in graph but show sig for combined sexes is the same with Fig 5. Also no values indicated in the Results. The authors state only higher/lower but no raw values.

**Response:** Thanks for your comment. Regarding Fig. 5, we have made the following revisions:

(1) Figure revision: The original Fig. 5 presented separate box plots for males and females (LA Male, LA Female, HA Male, and HA Female). However, the two-way ANOVA revealed no significant group-by-sex interaction. Therefore, we have simplified the figure to display only two groups, LA and HA, with data from both sexes combined. Significance markers directly indicate the comparison between LA and HA groups.
(2) Raw values in Results: We have added the actual mean concentrations (± SD) for each biomarker in the LA and HA groups, as requested. These values are currently included in the Results section (p14, lines 293–297).
(3) Updated figure legend: The legend has been revised to clarify that the significance markers refer to the main effect of alpha-amylase group (LA vs. HA) (p14, lines 301–303).

All changes are highlighted in red in the revised manuscript.

-Line364-369, stating Factor 1 or 2 makes it confusing. The authors could simply indicate what each factor is without stating the Factor number.

**Response:** Thanks for your comment. In the Results section, we have removed the potentially confusing “Factor 1”, “Factor 2”, and “Factor 7” labels and currently refer to these components by their full names: “subjective sleep quality”, “sleep latency”, and “daytime dysfunction”, respectively. All changes are highlighted in red in the revised manuscript (see revised manuscript, p12, lines 246–248).

-Well, the authors performed a lot of analysis and it's no surprise some must show significance but if one looks at the figure one would see how it is very unlikely that it would show real significance. This is especially the case with the Pearson correlation results. If one looks the graph for example cortisol vs amylase or melatonin vs amylase. even though the number is significant (howevery very low R at 0.14), one would not believe there is no correlation (S6 fig and S4 table).

**Response:** Thanks for your comment. We agree that although the Pearson correlations between alpha-amylase and IgA, cortisol, and MT reached statistical significance (p < 0.05), the correlation coefficients were very small (r = 0.14). Given the sample size of 262, even minimal effects may achieve statistical significance; however, this does not necessarily indicate biological or practical relevance. To avoid overinterpretation, we have revised the Results and Discussion sections as follows:

(1) Discussion section (correlation paragraph): α-amylase showed statistically significant but very weak positive correlations with IgA, cortisol, and MT, whereas a strong correlation was observed with lysozyme. MT was weakly positively correlated with IgA and cortisol, and weakly negatively correlated with lysozyme, consistent with prior findings. Given the large sample size, these weak correlations should be interpreted cautiously and not overemphasized as biologically strong relationships^33^. The text has been softened from “positively correlated” to “showed a statistically significant, while very weak positive correlation” (p20, lines 431–436). We no longer claim that these weak correlations support a strong relationship. Instead, we mention that the strong correlation between alpha-amylase and lysozyme (r = 0.52) represents the most robust finding, while other correlations are weak and may not be physiologically meaningful.
(2) Supplementary Fig. S6: The scatter plots show the data distribution; we have added a note in the figure that the low r values reflect minimal linear association despite statistical significance due to the large sample size (see Fig. S6).

All changes are highlighted in red in the revised manuscript.

-The Discussion could still be improved. For example, the intro regarding the significance of the study is mentioned in the first paragraph and repeated again in the fifth paragraph, interrupting the explanation of results.

**Response:** Thanks for your comment. We have removed the redundant paragraph that repeated the study’s significance, thereby making the Discussion more concise and logically coherent. All changes are highlighted in red in the revised manuscript (see revised manuscript, Discussion section, p17-24, lines 366-518).

-I do believe the article merits publication but the way it is presented doens't convince me very much. There is too much overclaim. And the writing should be improved. A lot of editing is needed.

**Response:** Thanks for your comment. To address the issues of “overstatement” and writing quality, we have systematically replaced strong or assertive language with more cautious, evidence-based phrasing. We have carefully edited the entire text to ensure that the language is clear, concise, and scientifically accurate. Redundant expressions and overly promotional language have been removed. We confirm that these revisions have significantly improved the scientific rigor and readability of the manuscript.

-Also I don't see how the quality of the figures improve much regarding intelligibility. but if the is acceptable for the journal I have no further comment regarding this point. I feel some of the supplementary figure should be presented as real figure (not just a supplement) while some could be supplementary figure (for example Fig 4) but it's just my opinion.

**Response:** Thank you for your comment. We have taken the following measures:

(1) Figure quality: All main figures have been regenerated at a higher resolution, and the font sizes of axis labels, legends, and data labels have increased. Supplementary figures have also been improved. We confirm that the improved figures meet the journal’s readability requirements.

(2) Figure placement: We have carefully considered your suggestion; however, we note that the journal accepted the current figure layout during the initial revision. To avoid delays in publication, we have decided to retain the existing layout. Nevertheless, we appreciate your insights and will take them into account in the future study.

We greatly appreciate the editor's and reviewers' time and effort in improving our manuscript, and we remain available for any further clarifications.
