# Supplementary material for: The hidden burden of college life: Stress, sleep, and salivary biomarkers in students—A cross-sectional study
Source: PLOS Ment Health. 2026 Sep 15;3(9):e0000717. doi: 10.1371/journal.pmen.0000717 (PMC13577347; doi:10.1371/journal.pmen.0000717)
Supplement: Peer review history

# Author comment: Author response to Decision Letter 0

**DOI:** 10.1371/journal.pmen.0000717.r001

**Transfer Alert**

This paper was transferred from another journal. As a result, its full editorial history (including decision letters, peer reviews and author responses) may not be present.

17 Dec 2025

# Peer review: Decision Letter 0

**DOI:** 10.1371/journal.pmen.0000717.r002

**Authors:** Karli Montague-Cardoso

26 Mar 2026

PMEN-D-25-00591

The Hidden Burden of College Life: Stress, Sleep, and Salivary Biomarkers in Students

PLOS Mental Health

Dear Dr. Xu,

Thank you for submitting your manuscript to PLOS Mental Health. After careful consideration, we feel that it has merit but does not fully meet PLOS Mental Health’s publication criteria as it currently stands. Therefore, we invite you to submit a revised version of the manuscript that addresses the points raised during the review process.

Please ensure that you fully address all of the points raised, which you can find at the end of this email.

Please submit your revised manuscript by Apr 24 2026 11:59PM. If you will need more time than this to complete your revisions, please reply to this message or contact the journal office at mentalhealth@plos.org. When you're ready to submit your revision, log on to https://www.editorialmanager.com/pmen/ and select the 'Submissions Needing Revision' folder to locate your manuscript file.

Please include the following items when submitting your revised manuscript:

- A letter that responds to each point raised by the editor and reviewer(s). You should upload this letter as a separate file labeled 'Response to Reviewers'.
- A marked-up copy of your manuscript that highlights changes made to the original version. You should upload this as a separate file labeled 'Revised Manuscript with Track Changes'.
- An unmarked version of your revised paper without tracked changes. You should upload this as a separate file labeled 'Manuscript'.

Guidelines for resubmitting your figure files are available below the reviewer comments at the end of this letter.

As the corresponding author, your ORCID iD is verified in the submission system and will appear in the published article. PLOS supports the use of ORCID, and we encourage all coauthors to register for an ORCID iD and use it as well. Please encourage your coauthors to verify their ORCID iD within the submission system before final acceptance, as unverified ORCID iDs will not appear in the published article. *Only*  the individual author can complete the verification step; PLOS staff *cannot*  verify ORCID iDs on behalf of authors.

We look forward to receiving your revised manuscript.

Kind regards,

Karli Montague-Cardoso

Staff Editor

PLOS Mental Health

Journal Requirements:

1. Please provide a detailed online Financial Disclosure statement. This is published with the article. It must therefore be completed in full sentences and contain the exact wording you wish to be published.

a) State the initials, alongside each funding source, of each author to receive each grant. For example: “This work was supported by the National Institutes of Health (####### to AM; ###### to CJ) and the National Science Foundation (###### to AM).”

b) State what role the funders took in the study. If the funders had no role in your study, please state: “The funders had no role in study design, data collection and analysis, decision to publish, or preparation of the manuscript.”

For more information, please go to our submission guidelines:

https://journals.plos.org/mentalhealth/s/submission-guidelines#loc-financial-disclosure-statement

2. Please ensure that the funders and grant numbers match between the Financial Disclosure field and the Funding Information tab in your submission form. Note that the funders must be provided in the same order in both places as well.

3. In the online submission form, you indicated that "The datasets used and/or analyzed during the current study available from the corresponding author on reasonable request.".

All PLOS journals now require all data underlying the findings described in their manuscript to be freely available to other researchers, either

a) In a public repository,

b) Within the manuscript itself, or

d) Uploaded as supplementary information.

This policy applies to all data except where public deposition would breach compliance with the protocol approved by your research ethics board. If your data cannot be made publicly available for ethical or legal reasons (e.g., public availability would compromise patient privacy), please explain your reasons by return email and your exemption request will be escalated to the editor for approval. Your exemption request will be handled independently and will not hold up the peer review process, but will need to be resolved should your manuscript be accepted for publication. One of the Editorial team will then be in touch if there are any issues.

For further assistance, you may go to: http://journals.plos.org/mentalhealth/s/data-availability

4. Please provide separate main figure files in .tif or .eps format only and ensure that all files are under our size limit of 10MB.

For more information about how to convert your figure files please see our guidelines: https://journals.plos.org/mentalhealth/s/figures

5. We have noticed that you have uploaded Supporting Information files, but you have not included a list of legends. Please add a full list of legends for your Supporting Information files before or after the references list.

6. Some material included in your submission may be copyrighted. According to PLOS’s copyright policy, authors who use figures or other material (e.g., graphics, clipart, maps) from another author or copyright holder must demonstrate or obtain permission to publish this material under the Creative Commons Attribution 4.0 International (CC BY 4.0) License used by PLOS journals. Please closely review the details of PLOS’s copyright requirements here: PLOS Licenses and Copyright. If you need to request permissions from a copyright holder, you may use PLOS's Copyright Content Permission form.

Please respond directly to this email or email the journal office and provide any known details concerning your material's license terms and permissions required for reuse, even if you have not yet obtained copyright permissions or are unsure of your material's copyright compatibility.

Potential Copyright Issues:

Figure 1: Please confirm whether you drew the images / clip-art within the figure panels by hand. If you did not draw the images, please provide (a) a link to the source of the images or icons and their license / terms of use; or (b) written permission from the copyright holder to publish the images or icons under our CC-BY 4.0 license. Alternatively, you may replace the images with open source alternatives. See these open source resources you may use to replace images / clip-art:

- https://commons.wikimedia.org

- https://openclipart.org/

7. If the reviewer comments include a recommendation to cite specific previously published works, please review and evaluate these publications to determine whether they are relevant and should be cited. There is no requirement to cite these works unless the editor has indicated otherwise.

8. Please review your reference list to ensure that it is complete and correct. If you have cited papers that have been retracted, please include the rationale for doing so in the manuscript text, or remove these references and replace them with relevant current references. Any changes to the reference list should be mentioned in the rebuttal letter that accompanies your revised manuscript. If you need to cite a retracted article, indicate the article’s retracted status in the References list and also include a citation and full reference for the retraction notice.

Additional Editor Comments (if provided):

[Note: HTML markup is below. Please do not edit.]

Reviewers' comments:

Reviewer's Responses to Questions

**Comments to the Author**

1. Does this manuscript meet PLOS Mental Health’s publication criteria?

Reviewer #1: Partly

Reviewer #2: Yes

**********

2. Has the statistical analysis been performed appropriately and rigorously?

Reviewer #1: Yes

Reviewer #2: I don't know

**********

3. Have the authors made all data underlying the findings in their manuscript fully available (please refer to the Data Availability Statement at the start of the manuscript PDF file)?

The PLOS Data policy

Reviewer #1: Yes

Reviewer #2: Yes

**********

4. Is the manuscript presented in an intelligible fashion and written in standard English?

Reviewer #1: Yes

Reviewer #2: Yes

**********

Reviewer #1: General information

- The journal requires double spacing.

- I recommend that authors use the same font and font size for the main text and the bibliography (main text: Times 12/bibliography: Calibri 14).

- To comply with the journal’s submission guidelines, the References section will need to be reformatted, mainly to adjust the authors’ punctuation and to organize the publication details in the correct chronological format.

- Materials and Methods? Instead of methods

- Ending section

The following elements are required, in order:

Acknowledgments

References

Supporting information captions (if applicable)

- Figure captions are inserted immediately after the first paragraph in which the figure is cited. Figure files are uploaded separately.

Tables are inserted immediately after the first paragraph in which they are cited.

Supporting information files are uploaded separately.

- Add short title no longer than 70 characters

- Fix bibliography format. Correct example Published articles

Hou WR, Hou YL, Wu GF, Song Y, Su XL, Sun B, et al. cDNA, genomic sequence cloning and overexpression of ribosomal protein gene L9 (rpL9) of the giant panda (Ailuropoda melanoleuca). Genet Mol Res. 2011;10: 1576-1588.

Abstract

- The abstract states that “90.4% experienced clinically significant stress.” However, when reviewing the results in the body of the manuscript, that 90.4% is broken down into ‘moderate’ (56.83%) and “high” (33.59%) stress. Labeling overall moderate stress as “clinically significant” in the abstract could be considered an overly strong or alarmist statement that does not accurately reflect the categorization of the PSS-14 scale detailed in the methods.

- The abstract indicates HPA axis hyperactivity by stating “cortisol↑.” While useful for summarizing, the main text details that students with high stress show an atypical diurnal pattern (lack of normal morning peak and an anomalous peak at 2:00 p.m., along with elevated nighttime levels). A simple “cortisol↑” misses the nuance that the actual finding is an alteration in the diurnal rhythm rather than a simple basal increase.

- The abstract is very competent and appealing to readers. Is necesary a minor adjustment in the wording about “clinically significant stress” to better align it with the results data,

Introduction

- Lines 57-60: I suggest that the authors ensure that all theoretical statements in the “Introduction” section are properly supported with their respective bibliographic references.

- Lines 76-112: This paragraph is very long (it occupies almost 36 continuous lines in the original manuscript). The density of medical acronyms and signaling cascades causes cognitive fatigue in the reader. I recommend that the authors strategically divide the paragraph or summarize the information.

- Lines 125-130: The final results of the study should not be revealed in the Introduction. This section is for stating the state of the art of the study idea.

- Lines 123-125: The paragraph states that the study proposes a “novel self-assessment paradigm” that allows for non-invasive quantification of physiology. The actual methodology of the study requires participants to donate 11 saliva samples over 24 hours under the supervision of technicians and in designated rooms. This is a rigorous experimental design, not a simple self-assessment. It is recommended to change the term to “novel non-invasive psychobiological profiling approach” or similar.

- Lines 139-150: Methodologically, it is not correct to describe the study in this section. I recommend that the authors delete this part or move it to the discussion section.

- In the last paragraph of the introduction, the text hypothesizes “IgA/LZM suppression.” However, the abstract and results of the article reveal that what was actually found was an activation/elevation of immunity in highly stressed students (IgA↑, lysozyme↑). I suggest revising the “Discussion” section to explicitly and clearly address why the authors expected immune suppression but ended up finding activation.

Methods

- Line 178: I suggest the authors change the verb tense to past tense: “Participants are permitted to maintain their regular eating...”

- The text states at the beginning that students were recruited between “March 3, 2023, and May 5, 2023” (a period spanning just over two months). However, a few lines below, the same paragraph concludes by stating that “Data collection occurred over three weeks.” I recommend that the authors define the exact period.

- Lines 198-201: What was the exact alpha level (e.g., 0.05)? What was the statistical power achieved (e.g., >80%)? What was the effect size detected?

- Line 196: The authors mention “A random selection of 285 students from the full sample was made for biomarker analysis,” but in the results section they mention “Totally, 262 salivary samples were collected at 3 PM and analyzed using ELISA.” How do the authors explain this difference?

Lines 217-219: I recommend that the authors remove the mention of the FSS and PSQI alphas from the PSS paragraph. Each paragraph should describe only its own assessment tool to maintain a logical reading flow.

Lines 241-242: I recommend that the authors clarify whether participants had to meet all criteria simultaneously (“and”) or only one of them (“or”).

Line 241: In the “Instruments and Data Collection” section, good sleep quality is defined as “PSQI ≤ 5.” However, this section states that to enter the Low Stress (LS) group, participants had to have “PSQI < 5.” If a student scores exactly 5 on the PSQI, do they qualify for the LS group or are they excluded? Mathematical notation should be perfectly consistent throughout the manuscript.

Line 253: I recommend that the authors use verbs in the past tense, given that these activities have already been carried out.

Lines 303-306: It is suggested to explicitly indicate which post hoc test was applied after the ANOVA (e.g., Bonferroni or other correction).

Lines 318-319: I suggest that the authors indicate "if the data were not normal, did they transform them logarithmically before applying ANOVA and Pearson? Or did they use nonparametric alternatives (such as Spearman's correlation or Kruskal-Wallis/Mann-Whitney tests)? I believe this should be clearly specified.

Results

- In one paragraph, the authors state that second-year students had significantly higher scores on the PSS compared to first-year students (p = 0.03). However, in the following paragraph, they conclude that “No significant variations in prevalence were identified across academic years (χ²=6.87, p=0.65).” Although mean scores may differ statistically without affecting the overall prevalence categorization, this distinction may not be entirely clear to readers. It would be helpful to include a brief clarifying statement indicating that, while raw mean scores varied (with second-year students showing higher values), the distribution across clinical categories (low, moderate, and high) did not differ significantly.

- Lines 401-402: The acronym MT is repeated in the title and IgA is omitted, even though it is also analyzed in this section.

- Lines 412.420 I recommend that the authors add the p-values to support the differences.

Discussion

- The discussion adequately integrates psychological and biological findings, which helps reinforce the impact of the study. However, a careful final review of the text is recommended, as there are some duplicate paragraphs that should be corrected.

Reviewer #2: This article examines the stress in undergrads by measuring many salivary biomarkers as well as questionnaires. This should provide insight into salivary biomarkers. The article merits publication; however, some points should be addressed first.

Title

-I'm not sure if "a cross-sectionaly study" should be added.

Abstract

-It would be nice to also note the p value when reporting r values.

Results

-The fonts in all the figures are very small and barely intelligible. It would be nice to adjust the make them more readable.

For example, the numbers are so tiny and just by not keeping decimal could make it more easily to read.

-Some values are in the range of 100,000 ng/ml. It would be more easily understandable if using ug/ml so the numbers are smaller amount.

-Fig2: It would be nice to also indicate which range of scores means mild/mod/severe

-PSQI in Fig 2 use score 7 which is different from the text that use 5. Please check

-Please check scores for FSS in Fig 2. I think there is some mistake. The % and score don't match with the text.

-For all figures, please use the symbol ≤ not <= as it's very difficult to read. Would also be nice to state HS LS groups in the x axes.

-Fig 3 and 5: It's impossible to tell which color represent what values as they are quite similar. There are 6 colors in the axes while in the figure there are 4. Maybe showing the box-plot like in Fig 2 would be easier to understand. Or should indicate the values in the text. ALso please avoid redundancy, like indicationg IgA (Immunoglobulin A), choose one.

-Fig 2E and 2F: it's a bit surprising that LS and HS groups had different PSQI and FSS since the box plot showed quite similar values. Please check.

-Pearson's correlations were actually very weak (for example, only r=0.14 for amylase vs IgA). It would be nice to show the scatter plot of these values in supplementary file.

-It would be nice to indicate the unit of the biomarkers values in figure legends or in the figures themselves.

-Fig 6 should really be adjusted as the current version is intelligible. It's too small. Actually the left and middle panels are not necessary as both are already shown in the right panels.

Discussion:

-It's very long and tedious to read. Some information is redundant or mentioned many times.

-It would also be very nice to make a paragraph shorter for readability.

**********

what does this mean?). If published, this will include your full peer review and any attached files.

**Do you want your identity to be public for this peer review?**  If you choose “no”, your identity will remain anonymous but your review may still be made public.

For information about this choice, including consent withdrawal, please see our Privacy Policy

Reviewer #1: No

Reviewer #2: No

**********

[NOTE: If reviewer comments were submitted as an attachment file, they will be attached to this email and accessible via the submission site. Please log into your account, locate the manuscript record, and check for the action link "View Attachments". If this link does not appear, there are no attachment files.]

# Author comment: Author response to Decision Letter 1

**DOI:** 10.1371/journal.pmen.0000717.r003

20 Apr 2026

Attachment Submitted filename: Response letter.docx

# Peer review: Decision Letter 1

**DOI:** 10.1371/journal.pmen.0000717.r004

**Authors:** Karli Montague-Cardoso

28 May 2026

PMEN-D-25-00591R1

The Hidden Burden of College Life: Stress, Sleep, and Salivary Biomarkers in Students — A Cross-Sectional Study

PLOS Mental Health

Dear Dr. Xu,

Thank you for submitting your revised manuscript to PLOS Mental Health. After careful consideration of the reviewer comments we invite you to submit another version of the manuscript that addresses the points raised during the review process. You will see from the comments raised that the reviewers felt that their points in the first round were not fully addressed. We require you to fully address the comments and may be unable to proceed further manuscript should you not do this. Thank you for your understanding.

Please submit your revised manuscript by Jul 26 2026 11:59PM. If you will need more time than this to complete your revisions, please reply to this message or contact the journal office at mentalhealth@plos.org. When you're ready to submit your revision, log on to https://www.editorialmanager.com/pmen/ and select the 'Submissions Needing Revision' folder to locate your manuscript file.

Please include the following items when submitting your revised manuscript:

- A letter that responds to each point raised by the editor and reviewer(s). You should upload this letter as a separate file labeled 'Response to Reviewers'.
- A marked-up copy of your manuscript that highlights changes made to the original version. You should upload this as a separate file labeled 'Revised Manuscript with Track Changes'.
- An unmarked version of your revised paper without tracked changes. You should upload this as a separate file labeled 'Manuscript'.

Guidelines for resubmitting your figure files are available below the reviewer comments at the end of this letter.

As the corresponding author, your ORCID iD is verified in the submission system and will appear in the published article. PLOS supports the use of ORCID, and we encourage all coauthors to register for an ORCID iD and use it as well. Please encourage your coauthors to verify their ORCID iD within the submission system before final acceptance, as unverified ORCID iDs will not appear in the published article. *Only*  the individual author can complete the verification step; PLOS staff *cannot*  verify ORCID iDs on behalf of authors.

We look forward to receiving your revised manuscript.

Kind regards,

Karli Montague-Cardoso

Staff Editor

PLOS Mental Health

Journal Requirements:

If the reviewer comments include a recommendation to cite specific previously published works, please review and evaluate these publications to determine whether they are relevant and should be cited. There is no requirement to cite these works unless the editor has indicated otherwise.

Additional Editor Comments (if provided):

[Note: HTML markup is below. Please do not edit.]

Reviewers' comments:

Reviewer's Responses to Questions

**Comments to the Author**

Reviewer #1: All comments have been addressed

Reviewer #2: (No Response)

**********

publication criteria?

Reviewer #1: Yes

Reviewer #2: Partly

**********

3. Has the statistical analysis been performed appropriately and rigorously?

Reviewer #1: Yes

Reviewer #2: I don't know

**********

4. Have the authors made all data underlying the findings in their manuscript fully available (please refer to the Data Availability Statement at the start of the manuscript PDF file)?

The PLOS Data policy

Reviewer #1: Yes

Reviewer #2: Yes

**********

5. Is the manuscript presented in an intelligible fashion and written in standard English?

Reviewer #1: Yes

Reviewer #2: No

**********

Reviewer #1: The authors complied with most of the requested guidelines regarding formatting, restructuring and methodological clarifications:

1. General format and structure

The manuscript was fully standardised in terms of formatting, using double spacing and 12-point Times New Roman font throughout all sections, including the references. Furthermore, the formal presentation of the article was enhanced by incorporating the methodological design into the title (“A Cross-Sectional Study”) and creating an appropriate short title of 68 characters. In the methodology section, the heading was correctly amended to “Materials and Methods”, in line with standard scientific nomenclature. Finally, the references were reorganised according to the Vancouver/PLOS format, correcting the chronological order and author punctuation, and removing residual metadata that remained in the document.

2. Abstract

The abstract was revised to improve the scientific accuracy of its statements. In particular, the wording relating to perceived stress was amended, replacing the phrase “clinically significant stress” with a more appropriate and objective description: “90.4% experienced moderate-to-high perceived stress”. Likewise, the finding associated with cortisol was reformulated with greater conceptual rigour, now described as “hypothalamic-pituitary-adrenal (HPA) axis dysregulation characterised by altered diurnal cortisol patterns”, thereby avoiding oversimplified interpretations. P-values were also included alongside correlation coefficients (r) in the main results reported in the abstract, thereby strengthening the statistical transparency of the summary.

3. Introduction

The introduction has been reorganised to improve its clarity and flow. The lengthy paragraph on signalling cascades, which made the text difficult to follow, has been split up and strategically summarised to make it easier to understand. Furthermore, premature findings and conclusions that were included in this section have been removed, thereby maintaining the structural coherence expected of a scientific introduction. Finally, the methodologically imprecise term ‘novel self-assessment paradigm’ was replaced by the expression ‘novel non-invasive psychobiological profiling approach’, which more accurately represents the methodological approach used in the study.

4. Materials and methods

The ‘Materials and methods’ section was strengthened in terms of both writing style and methodological rigour. Verbs were changed to the past tense to maintain narrative consistency, using expressions such as ‘Participants were permitted…’. The timeline of the study was also clarified, specifying that the recruitment process lasted two months, whilst the intensive data collection took place over three weeks. From a statistical perspective, the alpha significance level (0.05), statistical power greater than 80% and the expected effect size (Cohen’s d ≥ 0.5) were explicitly added. Furthermore, the use of the post-hoc LSD test following the ANOVA was detailed, as well as the application of Spearman’s correlations and logarithmic transformations for the analysis of non-normal data.

5. Results and Discussion

Relevant clarifications were incorporated into the results and discussion section to improve the interpretation of the findings. A specific explanation was added stating that, although there were variations in average scores across academic years, the categorisation of clinical prevalence remained largely unchanged. Furthermore, the discussion regarding the IgA/LZM markers was reorganised appropriately, moving the analysis of the paradoxical finding, where immune suppression was expected but activation was observed, to a more robust paragraph within the discussion. This new analysis proposes a transient response or an acute-over-chronic activation phenomenon as a possible explanation. Finally, the figures were optimised to improve the visualisation of the results: the former Figure 6, which was difficult to interpret due to its size, was divided into three separate figures (Figures 6, 7 and 8), and a supplementary appendix (S6 Fig) was also added with scatter plots to complement the analysis of the diurnal profiles.

Although the manuscript shows significant improvements and is generally of a high standard, there are still some areas that require further revision. In particular, certain corrections mentioned by the authors in their rebuttal letter have not been fully incorporated into the current version of the manuscript, and some methodological justifications could be strengthened to ensure greater clarity and consistency prior to formal acceptance.

1. Critical Discrepancy in PSQI Cut-off Points

A significant inconsistency remains in the definition of poor sleep quality. The methodological text sets the cut-off point as PSQI >5, whereas the caption for Figure 2 uses PSQI >7. In their response letter, the authors explicitly stated that this discrepancy had been corrected (“We thank the reviewer for identifying this discrepancy, which has been resolved accordingly”); however, upon reviewing the current version of the manuscript, I found that the problem persists. On page 16, lines 347–348, the caption for Figure 2 continues to state: “Participants with PSQI >7 were classified as having poor sleep quality”, contradicting the methodology described previously. I consider that this inconsistency must be corrected before acceptance, as it affects the internal coherence of the manuscript and reflects insufficient editorial review of the final version.

2. Inconsistency of Units in the Text vs. Figures.

I previously requested that the units of measurement shown in the figures be changed, replacing values expressed in ng/ml with more readable units, such as g/ml, in order to facilitate the visual interpretation of the data. In their response, the authors state that they made this change only in the text of the manuscript, but decided to retain the original units (“ng/ml” and “pg/ml”) in the heatmaps and boxplots, arguing that these correspond to the raw data obtained directly from the ELISA kits and that this would avoid potential conversion errors.

However, I consider that this justification does not adequately resolve the issue raised. Currently, the manuscript presents values such as “8.98 g/ml” in the text and “8980 ng/ml” in the figures simultaneously, which hinders comparative reading and may cause unnecessary confusion for the reader. From an editorial perspective, it is essential to maintain consistency in the units of measurement throughout the manuscript. I therefore recommend standardising the units used.

Reviewer #2: The authors have made effort to improve the manuscript. However, there are some issues of concern.

Actually, some of the issues have not been addressed even though the authors stated so.

-For example, Figure 2. There are still discrepancies between text and figure legend. Nothing changed.

In the text, PSQI was cutoff at 5 (Line 341) while in Figure legend PSQI cutoff was 7 (Line 348).

-The discrepancies regarding FSS values still remained. 51.02% = mod fatigue, 31.55%= severe fatigue (Line343-344). But in the Fig2C, FSS >5 is 51.02% and FSS 4-4.9 =31.55%.

-Fig 2 D-F showed graph as male female while the text explain difference between LS and HS groups withour differentiang sexes. The significant difference maker was on either male of female in the Fig which is misleading. Should clearly state significant difference compare with what group.

-This situation of sex in graph but show sig for combined sexes is the same with Fig 5. Also no values indicated in the Results. The authors state only higher/lower but no raw values.

-Line364-369, stating Factor 1 or 2 makes it confusing. The authors could simply indicate what each factor is without stating the Factor number.

-Well, the authors performed a lot of analysis and it's no surprise some must show significance but if one looks at the figure one would see how it is very unlikely that it would show real significance. This is especially the case with the Pearson correlation results. If one looks the graph for example cortisol vs amylase or melatonin vs amylase. even though the number is significant (howevery very low R at 0.14), one would not believe there is no correlation (S6 fig and S4 table).

-The Discussion could still be improved. For example, the intro regarding the significance of the study is mentioned in the first paragraph and repeated again in the fifth paragraph, interrupting the explanation of results.

-I do believe the article merits publication but the way it is presented doens't convince me very much. There is too much overclaim. And the writing should be improved. A lot of editing is needed.

-Also I don't see how the quality of the figures improve much regarding intelligibility. but if the is acceptable for the journal I have no further comment regarding this point. I feel some of the supplementary figure should be presented as real figure (not just a supplement) while some could be supplementary figure (for example Fig 4) but it's just my opinion.

**********

what does this mean?). If published, this will include your full peer review and any attached files.

**Do you want your identity to be public for this peer review?**  If you choose “no”, your identity will remain anonymous but your review may still be made public.

For information about this choice, including consent withdrawal, please see our Privacy Policy

Reviewer #1: No

Reviewer #2: No

**********

[NOTE: If reviewer comments were submitted as an attachment file, they will be attached to this email and accessible via the submission site. Please log into your account, locate the manuscript record, and check for the action link "View Attachments". If this link does not appear, there are no attachment files.]

# Author comment: Author response to Decision Letter 2

**DOI:** 10.1371/journal.pmen.0000717.r005

16 Jul 2026

Attachment Submitted filename: Response to reviewers.docx

# Peer review: Decision Letter 2

**DOI:** 10.1371/journal.pmen.0000717.r006

**Authors:** Karli Montague-Cardoso

11 Aug 2026

PMEN-D-25-00591R2

The Hidden Burden of College Life: Stress, Sleep, and Salivary Biomarkers in Students — A Cross-Sectional Study

PLOS Mental Health

Dear Dr. Xu,

Thank you for submitting your revised manuscript to PLOS Mental Health. The reviewers have found this to be much improved and it is almost ready for acceptance pending a few more minor edits as suggested by reviewer 2 below. Given the minor nature of these comments, we will assess the final round of revisions in-house as opposed to sending it back to reviewers again.

Please submit your revised manuscript by Sep 09 2026 11:59PM. If you will need more time than this to complete your revisions, please reply to this message or contact the journal office at mentalhealth@plos.org. When you're ready to submit your revision, log on to https://www.editorialmanager.com/pmen/ and select the 'Submissions Needing Revision' folder to locate your manuscript file.

Please include the following items when submitting your revised manuscript:

- A letter that responds to each point raised by the editor and reviewer(s). You should upload this letter as a separate file labeled 'Response to Reviewers'.
- A marked-up copy of your manuscript that highlights changes made to the original version. You should upload this as a separate file labeled 'Revised Manuscript with Track Changes'.
- An unmarked version of your revised paper without tracked changes. You should upload this as a separate file labeled 'Manuscript'.

Guidelines for resubmitting your figure files are available below the reviewer comments at the end of this letter.

As the corresponding author, your ORCID iD is verified in the submission system and will appear in the published article. PLOS supports the use of ORCID, and we encourage all coauthors to register for an ORCID iD and use it as well. Please encourage your coauthors to verify their ORCID iD within the submission system before final acceptance, as unverified ORCID iDs will not appear in the published article. *Only*  the individual author can complete the verification step; PLOS staff *cannot*  verify ORCID iDs on behalf of authors.

We look forward to receiving your revised manuscript.

Kind regards,

Karli Montague-Cardoso

Staff Editor

PLOS Mental Health

Journal Requirements:

If the reviewer comments include a recommendation to cite specific previously published works, please review and evaluate these publications to determine whether they are relevant and should be cited. There is no requirement to cite these works unless the editor has indicated otherwise.

Please review your reference list to ensure that it is complete and correct. If you have cited papers that have been retracted, please include the rationale for doing so in the manuscript text, or remove these references and replace them with relevant current references. Any changes to the reference list should be mentioned in the rebuttal letter that accompanies your revised manuscript. If you need to cite a retracted article, indicate the article’s retracted status in the References list and also include a citation and full reference for the retraction notice.

Additional Editor Comments (if provided):

[Note: HTML markup is below. Please do not edit.]

Reviewers' comments:

Reviewer's Responses to Questions

**Comments to the Author**

Reviewer #1: All comments have been addressed

Reviewer #2: All comments have been addressed

**********

publication criteria?

Reviewer #1: Yes

Reviewer #2: Yes

**********

3. Has the statistical analysis been performed appropriately and rigorously?

Reviewer #1: Yes

Reviewer #2: I don't know

**********

4. Have the authors made all data underlying the findings in their manuscript fully available (please refer to the Data Availability Statement at the start of the manuscript PDF file)?

The PLOS Data policy

Reviewer #1: Yes

Reviewer #2: Yes

**********

5. Is the manuscript presented in an intelligible fashion and written in standard English?

Reviewer #1: Yes

Reviewer #2: Yes

**********

Reviewer #1: All the suggestions were satisfactorily resolved by the others.

Reviewer #2: The authors have addressed all points raised.

However, there are some minor editorial issues that need to be addressed to improve the quality of the article.

-SAM is abbreviation. please write full name at first appearance.

-Line 166: Participants for diurnal sample collection

From the way it's written: 15 participants met LS criteria, and 18 met HS criteria. It seems strange that from 6xx subjects only 33 met the criteria. The LS is possible because all 3 criteria are required (PSS<28 + PSQI <5 + FSS <4) but for HS group only 1 criteria is required (either PSS >57 or PSQi >5 or FSS >5).

-Line 290: "MT concentrations showed positive correlations with concentrations of correlated positively with IgA."

Please correct.

-Mistake in Fig 7D and 7E. the graphs superimposed.

Also mistake in the Discussion (line413) "HS group showed elevated melatonin at 9:30"

Actually it's LS group, please check.

-Line 451: the blunted nighttime decline of amylase was not significantly different between HS and LS group, so cannot really conclude so.

**********

what does this mean?). If published, this will include your full peer review and any attached files.

**Do you want your identity to be public for this peer review?**  If you choose “no”, your identity will remain anonymous but your review may still be made public.

For information about this choice, including consent withdrawal, please see our Privacy Policy

Reviewer #1: No

Reviewer #2: No

**********

[NOTE: If reviewer comments were submitted as an attachment file, they will be attached to this email and accessible via the submission site. Please log into your account, locate the manuscript record, and check for the action link "View Attachments". If this link does not appear, there are no attachment files.]

# Author comment: Author response to Decision Letter 3

**DOI:** 10.1371/journal.pmen.0000717.r007

14 Aug 2026

Attachment Submitted filename: Response to reviewers.doc

# Editor report: Decision Letter 3

**DOI:** 10.1371/journal.pmen.0000717.r008

**Authors:** Karli Montague-Cardoso

19 Aug 2026

The Hidden Burden of College Life: Stress, Sleep, and Salivary Biomarkers in Students — A Cross-Sectional Study

PMEN-D-25-00591R3

Dear Dr. Xu,

We are pleased to inform you that your manuscript 'The Hidden Burden of College Life: Stress, Sleep, and Salivary Biomarkers in Students — A Cross-Sectional Study' has been provisionally accepted for publication in PLOS Mental Health.

Before your manuscript can be formally accepted you will need to complete some formatting changes, which you will receive in a follow up email. A member of our team will be in touch with a set of requests.

Please note that your manuscript will not be scheduled for publication until you have made the required changes, so a swift response is appreciated.

IMPORTANT: The editorial review process is now complete. PLOS will only permit corrections to spelling, formatting or significant scientific errors from this point onwards. Requests for major changes, or any which affect the scientific understanding of your work, will cause delays to the publication date of your manuscript.

If your institution or institutions have a press office, please notify them about your upcoming paper to help maximize its impact. If they'll be preparing press materials, please inform our press team as soon as possible -- no later than 48 hours after receiving the formal acceptance. Your manuscript will remain under strict press embargo until 2 pm Eastern Time on the date of publication. For more information, please contact mentalhealth@plos.org.

Thank you again for supporting Open Access publishing; we are looking forward to publishing your work in PLOS Mental Health.

Best regards,

Karli Montague-Cardoso

Staff Editor

PLOS Mental Health

***********************************************************

Reviewer Comments (if any, and for reference):
